# Blood Plasma Lipid Alterations Differentiating Psychotic and Affective Disorder Patients

**DOI:** 10.3390/biom15091296

**Published:** 2025-09-09

**Authors:** Anastasia Golubova, Elena Stekolshchikova, Anna Gareeva, Inessa Akhmerova, Ilgiz Timerbulatov, Valeria Zakurazhnaya, Daria Riabinina, Alexander Reznik, Anna Morozova, Denis Andreyuk, Georgiy Kostyuk, Daria Petrova, Anna Serkina, Philipp Khaitovich, Anna Tkachev

**Affiliations:** 1 Neuro Center, Moscow 121205, Russia; agolubova96@gmail.com (A.G.); petrova.msu@gmail.com (D.P.); anna.serkina@gmail.com (A.S.); khaitovich@gmail.com (P.K.); 2 Institute of Biochemistry and Genetics, Ufa Federal Research Center of the Russian Academy of Sciences, Ufa 450054, Russia; annagareeva@yandex.ru; 3 Department of Narcology, Russian Medical Academy of Continuous Professional Education of the Ministry of Healthcare of the Russian Federation, Moscow 125993, Russia; iftdoc@mail.ru; 4 Laboratory of Advanced Medical Projects, Kemerovo State University, Kemerovo 650043, Russia; 5 Department of Internal Medicine, Faculty of Medicine, Federal State Educational Institution of Highest Education Bashkir State Medical University of Public Health Ministry of Russian Federation, Ufa 450000, Russia; 6 Republican Clinical Psychiatric Hospital, Ministry of Healthcare of the Republic of Bashkortostan, Ufa 450069, Russia; akhmerova_inessa@mail.ru; 7 Department of Psychological Support and Clinical Psychology, University of Science and Technology, Ufa 450076, Russia; 8 F. A. Usoltsev Central Clinical Psychiatric Hospital, Ministry of Healthcare of the Moscow Region, Moscow 127083, Russia; 9 N. A. Semashko Scientific and Educational Institute of Clinical Medicine, Russian Medical University, Russian Ministry of Healthcare, Moscow 127006, Russia; 10 Mental-Health Clinic No. 1 Named After N. A. Alekseev, Moscow 117152, Russia; savva9806@yandex.ru (V.Z.); ryabinina.d98@yandex.ru (D.R.); a.m.reznik1969@gmail.com (A.R.); hakurate77@gmail.com (A.M.); denis.s.andreyuk@yandex.ru (D.A.); kgp@yandex.ru (G.K.); 11 Department of Psychiatry, Russian Biotechnological University, Moscow 125080, Russia; 12 Department of Basic and Applied Neurobiology, V. Serbsky Federal Medical Research Centre of Psychiatry and Narcology, Moscow 119034, Russia; 13 Economy Faculty, M.V. Lomonosov Moscow State University, Moscow 119991, Russia

**Keywords:** lipidomics, psychiatric disorders, biomarkers, schizophrenia, depression, mass spectrometry

## Abstract

Psychotic and affective disorders, including schizophrenia (SCZ) and depression (MDD), affect millions of people globally. The overlapping symptoms of these diseases and the lack of objective diagnostic tools could lead to misdiagnosis. Recent studies suggest that the analysis of plasma lipid levels may help to develop new diagnostic tools. In this study, we investigated the plasma lipidome of psychiatric patients and healthy controls to identify disease-specific lipid species. Using untargeted mass spectrometry, we profiled blood plasma lipids from 416 patients with common psychotic and affective disorders and 272 healthy individuals from two different cohorts. We observed lipidome alterations in SCZ and MDD consistent with earlier findings. In total, 144 lipids showed significant changes, with 107 of them being concordant across both disorders, and 37 being discordant. Lipids that differentiated SCZ from MDD were mainly triacylglycerols with polyunsaturated fatty acid residues decreased in MDD. In an additional group of 111 patients with bipolar, schizotypal, and schizoaffective disorders, these lipid markers suggested a trend toward separating psychotic and affective disorders. Furthermore, a logistic regression model trained on lipid data distinguished SCZ from MDD with an ROC AUC of 0.83. Taken together, these results suggest that blood lipid profiling may aid in the objective differentiation of psychotic and affective disorders.

## 1. Introduction

Severe mental disorders such as schizophrenia (SCZ) and depression (MDD) affect millions of people worldwide and have profound a societal impact [1]. These illnesses often present with complex manifestation: for example, SCZ includes hallucinations, delusions, disorganized behavior, and negative symptoms like reduced emotional expression and anhedonia [2], while MDD is marked by low mood, loss of interest, and insomnia [3]. Currently, diagnosis is symptom-based and relies on clinical evaluation using the Diagnostic and Statistical Manual of Mental Disorders (DSM-5) or the International Classification of Diseases 10 (ICD-10) criteria [2,4], requiring experienced psychiatrists. However, the heterogeneity of the symptoms, their temporal dynamics and the overlap between disorders complicate accurate diagnostics. For instance, both conditions often involve abnormal sleep, and many negative symptoms of SCZ also occur in MDD. Additionally, SCZ and MDD share genetic risk factors and exhibit similar cognitive and motor impairments [5,6]. These similarities contribute to frequent co-occurrence: MDD commonly emerges at various stages of SCZ [7], and psychotic symptoms can appear in MDD [8]. The subjective nature of symptom-based diagnostics may lead to mis- or overdiagnosis and inadequate treatment [9]. Thus, clinical diagnostics of SCZ and MDD could benefit from objective, laboratory-based tools such as molecular profiling.

The molecular pathogenesis of SCZ and MDD remains unresolved [10,11]. Although these diseases are primarily associated with brain dysfunction, they also induce systemic body changes, including alterations in blood composition. Omics studies have revealed consistent differences between patients and healthy individuals [12,13,14]. Lipidomics, in particular, is a promising tool for biomarker discovery [15,16,17]. Lipid alterations in blood plasma have been found to be similar in SCZ and MDD patients and mainly involved levels of glycerophospholipids and acylglycerols. Notably, both SCZ and MDD are associated with decreased abundance of plasmalogen phosphatidylcholines (PC-P) and phosphatidylethanolamines (PE-P), and ether-linked phosphatidylcholines (PC-O) [17,18,19,20,21], while triacylglycerols (TG) and acylcarnitines (CAR) tend to be elevated in SCZ and MDD patients [17,22,23,24,25,26]. These findings highlight shared molecular alterations in lipid metabolism across both disorders.

Although numerous studies report reproducible lipidomic changes in SCZ and MDD, evidence on the discriminative power of lipids between the disorders remains limited and inconsistent. Only a few works directly compared SCZ and MDD, with varying outcomes. For example, Oresic et al. linked elevated saturated and monounsaturated TG levels with SCZ, but not affective disorders [27]. Next, Wang et al. reported higher levels of ceramides (Cer) and lysophospholipids in SCZ compared to MDD [28]. Some studies included bipolar disorder (BPD) due to symptom overlap with SCZ and MDD. One research reports variations in fatty acyls across all three groups [29]. Meanwhile, Costa et al. observed altered glycerolipid and sphingolipid pathways in SCZ and BPD [30]. Finally, in a recent work, Tomasic et al. built a lipid-based model distinguishing MDD and BPD using levels of representative Cer, sphingomyelins (SM), phosphatidylcholines (PC), and TG, which also correlated with the manic symptom severity of BPD patients [31]. Overall, these findings suggest that lipidomics holds promise for differentiating mental illnesses, while highlighting the need for more studies involving larger sample sizes and multiple cohorts of patients representing different psychiatric disorders.

In this study, we examined the blood plasma lipid composition in individuals with diverse psychiatric diagnoses and unaffected controls, representing two independent cohorts from geographically and demographically distinct cities. Our primary objectives were to compare lipidomic alterations between patient groups within each cohort, evaluate the reproducibility of these changes, and identify lipid signatures distinguishing SCZ and SCZ-like disorders from MDD. Specifically, we aimed to uncover disease-specific lipid groups, assess their diagnostic potential, and suggest the biological mechanisms underlying these alterations. In addition, we sought to develop a mathematical classification algorithm to confirm the lipids’ ability to discriminate between different psychiatric conditions. To achieve these goals, we employed high-throughput mass spectrometry for comprehensive lipidome profiling, followed by a combination of statistical, clustering, and machine learning approaches. Our findings hold significant potential for insights into the molecular basis of these disorders and might show promise as a helpful tool in psychiatric clinics.

## 2. Materials and Methods

### 2.1. Subjects

Blood plasma samples were collected in two cities from psychiatric hospital in patients and unaffected controls (CTR). In total, 688 people (416 patients and 272 volunteers) were selected for comparative analysis based on the balance of age, sex, and body mass index (BMI) in each cohort (Table S1). The diagnoses were based on the criteria set according to ICD-10 after clinical interviews conducted by at least two independent specialists. The explored diagnoses included schizophrenia (F20), schizotypal disorder (F21), schizoaffective disorder (F25), bipolar affective disorder (F31), depressive episode (F32), and recurrent depressive disorder (F33). The latter two diagnoses, F32 and F33, were classified as “depression”, or MDD, in our analysis. All patients were treated according to standard treatment protocols in hospitals by taking antipsychotics or/and antidepressants and followed similar dietary recommendations. Exclusion criteria for both patients and control were age (<18 or >70 years old), intellectual disability, and severe somatic or neurological diseases that may affect a diagnosis of mental disorder, in line with ICD-10 diagnostic criteria. Individuals taking lipid-affecting drugs, such as statins, were excluded as well.

### 2.2. Study Design

**Figure 1:**
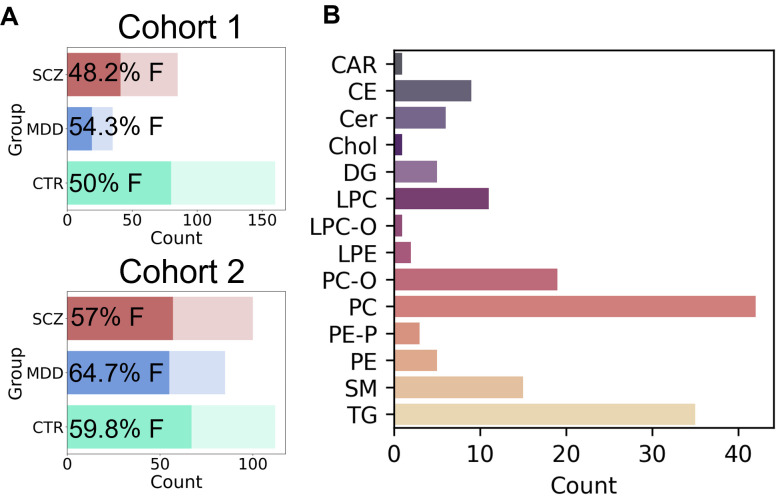
(**A**) The number of individuals in experimental groups: schizophrenia (SCZ), depression (MDD), and control (CTR) in Cohort 1 and Cohort 2. The percentage of females (F) is shown at each bar. (**B**) Count of detected lipid species distributed by classes: acylcarnitines (CAR), cholesterol esters (CE), ceramides (Cer), cholesterol (Chol), diacylglycerols (DG), lysophosphatidylcholines (LPC), ether LPC (LPC-O), lysophosphatidylethanolamines (LPE), phosphatidylcholines (PC), ether PC (PC-O), phosphatidylethanolamines (PE), plasmalogen PE (PE-P), sphingomyelins (SM), triacylglycerols (TG).

The blood plasma of people diagnosed with psychiatric disorders and CTR was collected in two cities; the groups will be referred to as Cohort 1 (C1) and Cohort 2 (C2). The two main psychiatric diagnoses presented in both cohorts were schizophrenia (SCZ) and major depressive disorder (MDD). Specifically, the collected samples included 160 CTR for C1 (age 34 ± 1, 50% female, Figure 1A, top) and 112 for C2 (age 32 ± 1, 59.8% female, Figure 1A, bottom), 85 SCZ patients for C1 (age 33 ± 1, 48.2% female, Figure 1A, top) and 100 SCZ patients for C2 (age 40 ± 2, 57% female, Figure 1A, bottom), and 35 MDD patients for C1 (age 33 ± 3, 54.3% female, Figure 1A, top) and 85 MDD patients for C2 (age 41 ± 2, 64.7% female, Figure 1A, bottom). In total, there were 280 and 297 samples for C1 and C2, respectively, including SCZ, MDD, and CTR groups. The groups inside the cohorts were balanced in terms of age and BMI (Table S1). Furthermore, all groups had a balanced female-male ratio with a small female predominance for the MDD patient groups (Figure 1A, Table S1).

Lipids extracted from the samples were analyzed using untargeted direct infusion mass spectrometry (DIMS), including the MS2 fragmentation step for reliable feature annotation. In total, we detected and annotated 155 lipids from 13 biochemical classes in the intersection of both datasets (Figure 1B). Among them, PC and TG, representing essential parts of lipoproteins, expectedly showed the highest number detected of lipid compounds (42 and 35, respectively).

### 2.3. Sample Collection

Both patients and control individuals were required to fast before blood collection for at least eight hours. Peripheral venous blood was collected in the morning using standard protocol. Briefly, 6 mL of blood from each patient/volunteer was collected into a vacutainer plastic tube (BD, Franklin Lakes, NJ, USA) containing anticoagulant K2EDTA and centrifuged within 30 min from sample collection for 15 min at 1500 rpm at room temperature (20–25 °C). After, a total of 500 μL of supernatant was placed in Eppendorf tubes and stored at −80 °C prior performing the experiments.

### 2.4. Lipid Extraction

Prior to the lipid extraction procedure, plasma samples were randomized to avoid any sample-order-related bias. Lipids were extracted using modified Matyash method [32]. An aliquot of 250 μL of water was added to 20 μL of plasma, followed by the addition of 1300 μL of cold mixture methyl tert-butyl ether (MTBE):methanol (7:2, v:v). After ultrasound exposure, shaking, and centrifugation, 1000 μL of upper phase was collected to a separate tube and dried under reduced pressure (20 Pa) at 30 °C (Concentrator plus, Eppendorf, Hamburg, Germany). Dried pellets were stored until the analysis at −80 °C. On the day of mass spectrometry measurements, pellets were reconstituted in a 200 μL mixture of isopropanol:methanol:chloroform (4:2:1; v:v:v) and diluted 5-fold with the same mixture using 9.5 mM ammonium formate as an additive.

### 2.5. MS Data Acquisition

Mass spectrometry analysis was performed in a positive mode using a QExactive mass spectrometer (Thermo Fisher Scientific, Waltham, Massachusetts, USA) equipped with a heated electrospray ionization source. Samples were introduced by flow injection using a Waters Acquity UPLC chromatograph (Waters, Manchester, UK). Each mass spectrometry experiment consisted of full scan events and subsequent data-independent acquisition (DIA). The mobile phase consisted of a 7.5 mM ammonium formate solution in isopropanol:methanol:chloroform (4:2:1; v:v:v) [33]. One run duration time was 3 min.

Quality control samples (QC) were made from pooled aliquots of the first 96 samples in the batch. QC samples were inserted after every 12 samples to account for batch effects and technical reproducibility and in the beginning of each batch to allow for system equilibration. For more details concerning the experimental part, see Supplementary Materials.

### 2.6. Lipid Identification and Data Post-Processing

Raw files were converted to mzXML format with PeakStrainer (2017) and then loaded to LipidXplorer software [34,35] (v. 1.2.8.1) using the import settings specified in Supplementary Materials. Lipid identification was based on Molecular Fragmentation Query Language (MFQL) scripts downloaded from the paper [33] along with customized MFQL files. The following lipid classes were included in the analysis: acylcarnitines (CAR), lysophosphatidylcholines (LPC), ether LPC (LPC-O), lysophosphatidylethanolamines (LPE), sphingomyelins (SM), triacylglycerols (TG), diacylglycerols (DG), phosphatidylcholines (PC), ether PC (PC-O), phosphatidylethanolamines (PE), plasmalogen PE (PE-P), cholesterol (Chol), cholesterol esters (CE), and ceramides (Cer). The table with feature intensities was run through customized R (v. 4.4.1) and Python (v. 3.12) scripts and included blank and QC filtrations and batch correction. For more details, see Supplementary Materials.

### 2.7. Statistical Analysis

Statistical analysis was performed following log_2_-transformation of mass spectrometry signal intensities, hereafter referred to as lipid abundances (intensities). Log_2_-transformed fold changes (FC) were defined as the difference in mean lipid abundances between two groups. Group-wise comparisons were conducted using unpaired two-tailed Student’s *t*-test; *p*-values (*p*) were adjusted (adj.) for multiple testing using the Benjamini-Hochberg procedure (BH); lipid differences with adj. *p* < 0.05 were considered as statistically significant. Pearson correlation (r) was used for correlation analysis. Class-level lipid shifts were assessed with one-sample Wilcoxon signed-rank test by comparing within-class FC (SCZ or MDD vs. CTR) to zero. K-means clustering (number of clusters was set to 6, maximum number of iterations was set to 1000) was applied to lipids significantly altered in either disorder vs. CTR (144 lipids in total). The Mann-Whitney U-test was utilized to compare clusters between SCZ and MDD to identify “concordant” and “discordant” groups of lipids. FC values were smoothed via sliding average of three consecutive values. Principal component analysis (PCA) was performed using SCZ, MDD, and CTR samples from both cohorts together using the lipids from “concordant” and “discordant” clusters to estimate the group distribution. For PCA biplots the loading scores of lipids were used.

Lipid class enrichment within clusters was evaluated using the hypergeometric test. Differences in TG double bond counts across “concordant” and “discordant” clusters were assessed via two-sided Fisher’s exact test. Partial least square discriminant analysis (PLS-DA) was performed on the intensities of the MDD-SCZ lipid panel (20 lipid) separately on each cohort to assess the group separation. Principal component analysis (PCA) was performed similarly on the intensities of MDD-SCZ lipid panel (20 lipids), and the first principal component (PC1) was used to differentiate diagnostic groups. The pairwise differences in PC1 distributions were estimated with the Kolmogorov-Smirnov (KS) test.

### 2.8. Prediction Classification Model

Logistic regression models for SCZ-CTR and MDD-CTR differentiation were constructed using CTR (label 0) and SCZ or MDD samples (label 1) and all 155 annotated lipids. LASSO (L1) regularization (hyperparameter C = 1 for SCZ-CTR model and C = 100 for MDD-CTR model) was used to select the most important features. Five-fold cross validation was utilized to prevent overfitting and assess the model performance. The performance of the models was estimated using the area under the receiver operating characteristic (ROC AUC) and F1-score. In a similar way, the logistic regression classification model for MDD/SCZ differentiation was constructed using SCZ (label 0) and MDD (label 1) samples from both cohorts, with 206 samples in total (165 in the training set and 41 in the test set; the exact proportions for each cohort are given in Table S2) and all 155 annotated lipids. Hyperparameter C = 1 was used for regularization. All the manipulations were performed using the modules of the *sklearn* Python package.

All the data processing, statistical manipulations, and pictures were performed or created using R and Python tools. The pictures were aligned in Inkskape and Microsoft PowerPoint.

## 3. Results

### 3.1. Blood Plasma Lipidome Alterations in Schizophrenia and Depression Patients

**Figure 2:**
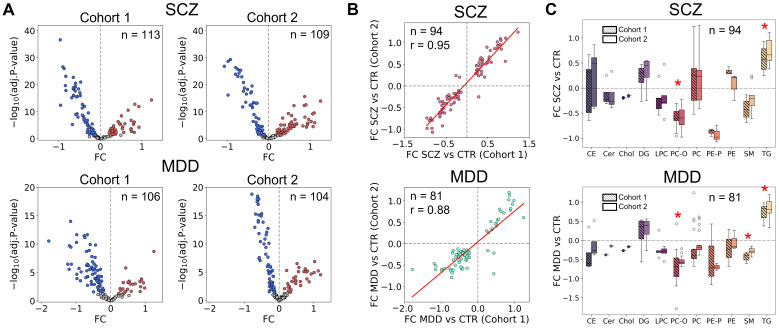
(**A**) Volcano plots for SCZ and MDD vs. CTR for Cohorts 1 and 2. Significantly altered lipids are red (for the increased ones) and blue (for the decreased ones), and n stands for the number of significantly altered lipids. The x-axis depicts the difference between each lipid log_2_-transformed intensity of SCZ or MDD and CTR (FC), and the y-axis depicts − log_10_ (adj. *p*-value) for *t*-test (lipid intensity of SCZ or MDD vs. CTR). (**B**) Correlations of log_2_-transformed FC in lipid abundances for SCZ or MDD against CTR between Cohort 1 and Cohort 2, where r stands for the Pearson correlation coefficient, and n stands for the number of intersected lipids. Red line depicts the linear regression line. (**C**) Boxplots showing the log_2_-transformed FC in lipid abundances for SCZ or MDD against CTR for the intersected lipids from both cohorts distributed by classes. Red asterisks above depict the pairs of boxplots where FC SCZ or MDD vs. CTR of lipids within a class showed a significant difference from 0 (one-sample Wilcoxon test, adj. *p* < 0.05).

We first assessed lipid abundance differences between SCZ or MDD patients and CTR using two-sided unpaired *t*-tests with BH correction. In each cohort and each disease, over 100 significantly altered lipids were identified: 113 (SCZ) and 106 (MDD) in C1; and 109 (SCZ) and 104 (MDD) in C2 (adj. *p* < 0.05; Figure 2A; Tables S3–S6). Most of the significant lipids overlapped across cohorts (94 lipids in intersection for SCZ and 81 for MDD). The FC of altered lipids showed strong inter-cohort correlation (Pearson correlation between the disease and CTR FC of each cohort, SCZ: *r* = 0.95, *p* = 1.4 × 10^−49^; MDD: *r* = 0.87, *p* = 5.3 × 10^−26^; Figure 2B), demonstrating the reproducibility of the changes. At the lipid class level, TG were elevated, and PC-O decreased in both disorders, while SM were significantly reduced only in MDD (one-sample Wilcoxon test, adj. *p* < 0.05; Figure 2C, bottom, Tables S7 and S8). The strength and amplitude of changes between SCZ-CTR and MDD-CTR correlated well within cohorts (Pearson correlation between SCZ-CTR and MDD-CTR FC, C1: *r* = 0.68, *p* = 5.9 × 10^−21^; C2: *r* = 0.94, *p* = 7.1 × 10^−67^; Figure S1), which additionally confirmed the similarity of alterations in human plasma during these disorders.

**Figure 3:**
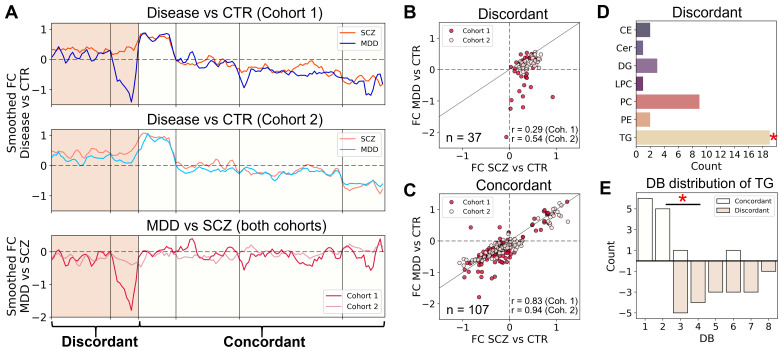
(**A**) K-means clustering analysis of log_2_-transformed FC between SCZ or MDD vs. CTR for both cohorts. First two graphs depict smoothed FC of disease vs. CTR for Cohort 1 and Cohort 2 datasets in the order of lipids according to the k-means analysis result, and vertical lines indicate the borders of clusters. The last plot shows smoothed FC of MDD vs. SCZ for cohorts. The left colored part of the plots highlights the clusters different between MDD and SCZ (discordant), and the right part shows similar clusters (concordant). (**B**) Correlations of FC in lipid abundances for SCZ or MDD vs. CTR for all lipids in the discordant clusters. (**C**) Correlations of FC in lipid abundances for SCZ or MDD vs. CTR for all lipids in the concordant clusters. For B and C, black lines are the diagonals of I and III quadrants; n stands for the number of lipids in the discordant or concordant clusters; and r stands for the Pearson correlation coefficient for both cohorts (Coh. 1 or Coh. 2). (**D**) Number of lipids of the discordant clusters distributed by classes. Red asterisk depicts the significance (adj. *p* < 0.05) of the hypergeometric test for enrichment of a particular lipid class in the discordant clusters among lipids of this class in all clusters. (**E**) Distribution of the double bonds (DB) among the TG included in the concordant and discordant clusters. Red asterisk depicts the significance (*p* < 0.05) of the Fisher’s exact test of DB number in TG of discordant vs. concordant lipid groups.

To further explore similarities and differences between lipidome alterations in SCZ and MDD and assess the consistency of the effects in two datasets, the disorder-against CTR FC of 144 altered lipids in at least one cohort were clustered via k-means, resulting in six clusters (Figure S2, Table S9). Of them, four clusters (107 lipids, 74% out of 144) showed similar behavior in SCZ and MDD (“concordant” group; Mann–Whitney U-test between FC of clusters for SCZ and MDD, adj. *p* > 0.05; Figure 3A, Table S10), while two clusters (37 lipids) displayed significantly different FC patterns (“discordant” group; adj. *p* < 0.05). This was reflected in the correlation analysis: the correlation of SCZ-CTR and MDD-CTR FC was strong for the “concordant” lipids (C1: *r* = 0.83, *p* = 5.6 × 10^−29^; C2: *r* = 0.94, *p* = 2.3 × 10^−52^), but weaker for the “discordant” ones (C1: *r* = 0.29, *p* = 0.078; C2: *r* = 0.54, *p* = 0.0006; Figure 3B,C). To additionally estimate the group distribution for “concordant” and “discordant” lipids, PCA analysis was performed separately on these two sets of lipid species. From the PCA biplot (Figure S3), it can be seen that for “concordant” lipids, all the disorders are grouped together and are separated from the control, but for the “discordant” group, they are separated from each other as well.

Biochemically, the “discordant” clusters were enriched in TG (hypergeometric test, adj. *p* = 0.000048; Figure 3D, Table S11) and showed a different number of total double bonds (DB) within fatty acid (FA) residues of TG compared to the “concordant” group. There was a significant shift in TG FA residues’ composition toward polyunsaturated fatty acids (PUFAs), with more than 4 DB in the “discordant” cluster lipids (Fisher’s exact test, *p* = 0.000297; Figure 3E, Table S12). Meanwhile, PC-O lipids were enriched in the “concordant” clusters (adj. *p* = 0.031; Table S13), confirming that this class is significantly affected in SCZ and MDD in comparison to a healthy state.

**Figure 4:**
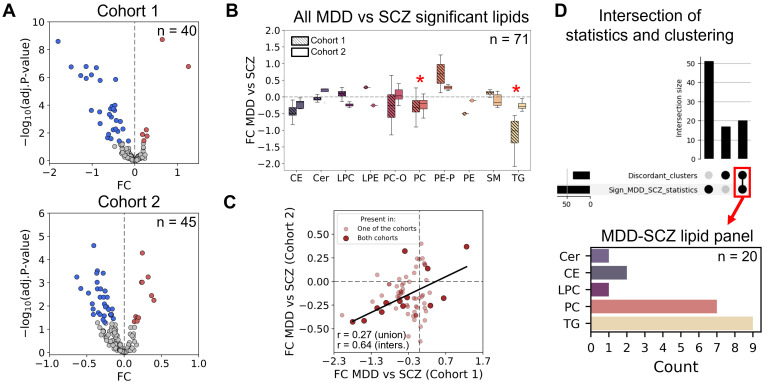
(**A**) Volcano plots for MDD vs. SCZ for Cohorts 1 and 2. Significantly altered lipids are red (for the increased ones) and blue (for the decreased ones); n stands for the number of significantly altered lipids; x-axis depicts the difference between each lipid log_2_-transformed intensity of MDD and SCZ (fold change, FC); and y-axis depicts −log_10_ (adj. *p*-value) for *t*-test (lipid intensity of MDD vs. SCZ). (**B**) Boxplots showing the log_2_-transformed FC in lipid abundances for MDD against SCZ of all the statistically significant lipids from both cohorts distributed by classes. Red asterisks above depict the pairs of boxplots where FC MDD vs. SCZ of lipids within a class showed a significant difference from 0 (one-sample Wilcoxon test, adj. *p* < 0.05). (**C**) Correlations of log_2_-transformed FC in lipid abundances for MDD against SCZ between Cohort 1 and Cohort 2. The lipids found to be significant in only one of the cohorts are depicted in light red color, while the intersection of significant lipids is shown in dark red points; r stands for the Pearson correlation coefficients (“union” for the all significant lipids in both cohorts (n = 71); and “inters.” for the intersection of significant lipids (n =14)). Black line depicts the linear regression line. (**D**) UpSet plot for statistically significant lipids between MDD and SCZ (“Sign_MDD_SCZ_statistics”) and those belonging to “discordant” clusters (“Discordant_clusters”). The intersection is shown in the red square (MDD-SCZ lipid panel). The bottom count plot shows the distribution of this panel by lipid classes.

We then directly compared lipid abundance between the MDD and SCZ groups in two cohorts. A total of 40 (C1) and 45 (C2) lipids were significantly altered between the disorders (unpaired two-sided *t*-test, adj. *p* < 0.05; Figure 4A, Tables S14 and S15), with most showing lower levels in MDD (33 decreased lipids out of 40 in C1 and 34 out of 45 in C2; Figure 4A,B). Fourteen lipids changed in the MDD-SCZ comparison that overlapped between the cohorts. FC values across 71 lipids significantly changed in at least one cohort, as well as the 14 overlapping ones, which were positively correlated between the cohorts (all altered lipids: *r* = 0.27, *p* = 0.023; overlap: *r* = 0.64, *p* = 0.012; Figure 4C), confirming the robustness of the shifts in lipid levels. Moreover, since some works report sex-specific differences in the lipidome for psychiatric disorders, including SCZ and MDD [36,37], we assessed the effect of these 71 significant lipids separately on group averages for males and females, respectively (the exact number of male/female samples in each cohort can be found in the Table S16). Correlation for FC between averaged values across two cohorts for males and females was fairly good (*r* = 0.7, *p* = 1.6 × 10^−11^, Figure S4A). The profiles were generally similar, however, the sex-mixed effects were better reproduced for women, especially for TG (Figure S4B, Table S17). Interestingly, while performing the statistical testing separately on males and females, there were more significantly altered lipids in females (*n* = 87) than in males (*n* = 26) and inter-cohort reproducibility was worse for males (unpaired two-sided *t*-test, adj. *p* < 0.05; Figure S5A, Table S18). In addition, all the lipids altered in males had been found in 71 significant lipids in the mixed-sex analysis, but for females, 30 lipids with predominant PC and TG were specific (Figure S5B, Table S18). Nevertheless, the sample size was not enough to discuss the sex-specific differences in detail during this study, and this may be continued in future research.

For further analysis, we focused on 20 lipids from the “discordant” clusters that also statistically differed between MDD and SCZ in at least one cohort in the sex-mixed analysis (“MDD-SCZ lipid panel”; Figure 4D). The lipid panel included mainly TG enriched in PUFA residues (Table S19), specifically with linoleic (18:2) and arachidonic (20:4) acids, as the most abundant PUFAs. This is in agreement with the DB profile of the “discordant” clusters, which demonstrated enrichment with PUFAs-containing TG (Figure 3D,E and Figure S6).

### 3.2. Psychotic and Affective Disorders Difference

**Figure 5:**
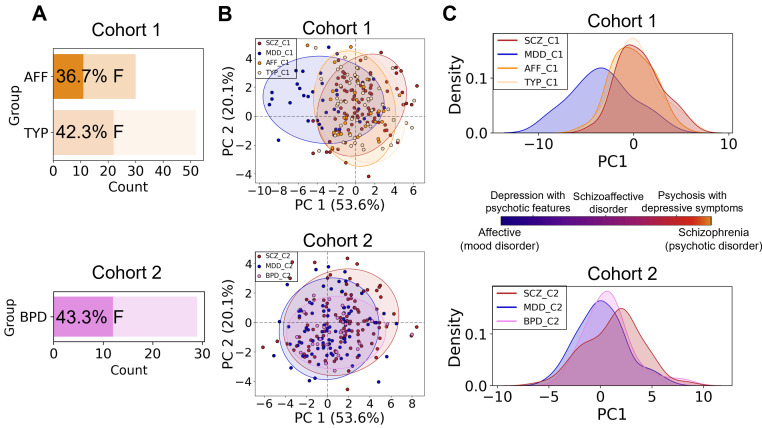
(**A**) The number of individuals in the experimental groups: schizoaffective (AFF) and schizotypal (TYP) in Cohort 1 and bipolar (BPD) in Cohort 2. The percentage of females (F) is shown on each bar. (**B**) Illustration of principal component analysis (PCA) for the species included in MDD-SCZ lipid panel performed on both cohorts together. Points corresponding to the different diseases are outlined in colored ellipses. The borders of the ellipses depict the area inside which the points of that group are found with 95% confidence. On the x-axis, the first principal component (PC1) is plotted, while on the y-axis, the second principal component (PC2) is plotted with the percentage of variation explained by this component in the brackets. (**C**) Kernel density estimation (KDE) of the first components (PC1) of PCA analysis in each cohort. The scale in the middle with symptom gradient is the modified scale of psychotic and affective disorders taken from [38]. C1 in the legends at (**B**,**C**) stands for “Cohort 1”, and C2 stands for “Cohort 2”.

In addition to SCZ and MDD, C1 included 52 patients with schizotypal (TYP) (age 31 ± 2, 42.3% female) and 30 patients with schizoaffective (AFF) (age 34 ± 3, 36.7% female) disorder, while C2 contained 29 patients with bipolar (BPD) (age 34 ± 4, 43.3% female) disorder (Figure 5A; Table S1). As lipidome data for all groups were acquired in the same randomized experiment, we evaluated whether the 20 lipids of the MDD-SCZ panel could also distinguish these additional diagnoses.

We first performed supervised clustering analysis based on the lipid abundances from the MDD-SCZ panel to estimate the groups’ separation and their distribution, including all the diseases and healthy controls. The groups were partially separated, in particular, CTR, MDD, and SCZ-like diseases for the first cohort, and CTR, SCZ, and MDD+BPD for the second cohort (Figure S7A). Then, PCA was performed based on the same lipids to assess the group separation in an unsupervised manner. The relative positions of the groups were similar to those obtained for PLS-DA (Figure S7B). PCA based on lipid abundance from the MDD-SCZ panel revealed clear separation of SCZ and MDD patients along PC1 in both cohorts (Figure 5B,C). In C1, SCZ-like disorder patients (TYP and AFF) clustered with SCZ and were significantly distinct from MDD (KS test, adj. *p* < 0.05 for TYP-MDD and AFF-MDD comparisons; Table S20). In C2, BPD patients separated from SCZ and aligned with MDD (KS test, adj. *p* < 0. 05 for BPD-SCZ comparison; Table S20). These results suggest that plasma levels of the selected lipid subgroup may support broader classification of psychotic SCZ-like (SCZ, AFF and TYP) and affective/mood (MDD and BPD) disorders.

### 3.3. MDD-SCZ Classification Model

As a first step and proof of concept, we constructed two disease-control models (SCZ-CTR and MDD-CTR), for which good separation has been reported previously. The metrics were expectably good for both models, and F1-scores exceeded 0.8 for both training and test datasets (Tables S21 and S22). However, the focus of the present study lied in the SCZ-MDD differentiation. To assess the predictive potential of blood lipidome for distinguishing SCZ and MDD, we built a logistic regression model using balanced, randomly selected samples from both cohorts to minimize the technical batch effect influence on the model performance and class imbalance (Table S2). All 155 detected lipids were included to avoid feature selection bias. The algorithm identified 45 lipids as major contributors, with PC and PC-O as dominant biochemical classes (Figure S8). Performance classification metrics were as follows: macro F1-scores of 0.97 (training dataset) and 0.74 (test dataset), and ROC AUC of 0.83 (Table S23, Figure S9A). Probability scores showed clear separation between SCZ and MDD (Figure S9B). The model was additionally estimated using only one cohort as the test set. The metrics appeared to be better for Cohort 1 than for Cohort 2 (macro average F1-score of 0.87 and 0.60 for Cohort 1 and 2, respectively; Table S24). The algorithm was then applied for classification of TYP, AFF, and BPD samples. TYP and AFF probability scores were skewed toward SCZ, while BPD showed a more variable distribution (Figure S10).

## 4. Discussion

We recruited patients and healthy controls from two hospitals in different cities to assess inter-cohort reproducibility of blood plasma lipidome alterations, particularly those distinguishing SCZ and MDD. Over 100 lipids significantly differed between patients and controls in each cohort, consistent with prior studies showing lipidomic differences in psychiatric disorders [17,30]. Comparing cohorts, we observed high reproducibility of the disorder-associated lipid changes with the Pearson correlation coefficients = 0.95 for SCZ and 0.88 for MDD, exceeding that in other multi-cohort studies [17,23]. Direct SCZ-MDD comparisons yielded reproducibility values of 0.64 (intersection) and 0.27 (union), still within acceptable range, with the lower correlation likely due to weaker SCZ-MDD lipidomic contrast compared to disorder-control differences.

Although SCZ and MDD are classified as distinct psychiatric disorders, they share many symptoms and exhibit overlapping genetic risk loci [39,40]. Correspondingly, most blood plasma lipidome alterations reported for SCZ and MDD also match. In our study, about three quarters of the disorder-associated lipid changes, mainly involving TG and PC-O, were similarly altered in both conditions. This aligns with the previous findings showing consistently elevated TG levels, playing an important role in energy storage, in both drug-naïve and medicated SCZ and MDD patients [21,22,23,24,27,41,42]. Conversely, membrane ether glycerophospholipids, particularly PC-O and PE-O, and plasmalogens (PC-P, PE-P) were decreased in both disorders [18,19,20,21,22,43]. We observed an evident decline in specific PC-O and PE-P species in the blood plasma of both SCZ and MDD patients (Figure 2C). As key components of cell membranes, ether phospholipids influence membrane integrity and dynamics [44]. Their shared dysregulation may reflect molecular mechanisms underlying shared symptom manifestations in these disorders.

While blood plasma lipidome alterations similar between SCZ and MDD are robustly observed, disorder-specific differences remain inconsistent. Among the few relevant works, Wang et al. reported elevated lysophospholipids in SCZ but not in MDD [28], and Yin et al. proposed several carnitines as potential biomarkers [45]. In our research, we identified 37 lipids that reproducibly differentiated SCZ and MDD across two cohorts, with 20 of them significantly changed in direct disease comparison (MDD-SCZ lipid panel). Biochemically, these 20 lipids from the panel were predominantly TG with PUFA residues, particularly containing linoleic (18:2) or arachidonic (20:4) ω-6 residues and showing lower levels in MDD than in both CTR and SCZ. Additionally, seven PC from the panel were decreased in MDD, six of which also contained highly unsaturated fatty acid residues. While there are no current reports directly associating PUFAs-containing TG or PC and MDD, several studies have presented results aligning with our observation. Oresic et al. observed increased saturated and monounsaturated TG specifically in SCZ [27] in comparison to other disorders, while Tomasik et al. identified PUFAs-containing TG and PC as important for distinguishing MDD and BPD, including TG 56:6, TG 53:4, PC-O 36:5, and PC 32:3 [31]. Interestingly, both of these TG compounds, as well as PC 32:3, were among the MDD-SCZ distinctive lipids found in our study.

PUFAs are vital components of the brain, found in esterified form in phospholipids and CE of neuronal cell membranes and forming 25–30% of lipid FA tails [46], but they are also found as unesterified free fatty acids. They are delivered via blood plasma lipids, such as TG, PC, and CE, which form lipoprotein complexes, and LPC, which can circulate through the blood-brain barrier [46,47,48]. In the brain, PUFAs support neurogenesis, neurotransmission, and synaptic function, and modulate neuroinflammation [46,49]. Accordingly, PUFAs levels and metabolism are implicated in psychiatric disorders, particularly MDD [50,51]. Reduced plasma ω-3 PUFAs levels have been reported in this disorder [52,53,54], potentially taking a particular role in the upregulation of serotonin receptors 5-HT2—one of the suggested pathological mechanisms in the serotonin hypothesis of MDD [55,56]. Besides, ω-3 supplementation shows therapeutic potential in unipolar and bipolar depression [50,53]. Furthermore, altered ω-3 and ω-6 PUFAs levels have also been observed in SCZ, especially in red blood cells [57,58,59] and in post-mortem brains of SCZ and MDD patients, likely due to increased phospholipase A2 activity or dietary deficiency [60,61]. Moreover, a recent study performed by our laboratory revealed the significant correlation between self-reported Hospital Anxiety and Depression Scale (HADS-D) scores, which show the severity of MDD symptoms, and certain lipid abundances in the cohort of volunteers [62]. Most of the lipids negatively correlated with HADS-D scores were ether phospholipids containing PUFAs. TG 58:8 also had negative association with MDD symptoms. Taken together, this may suppose the specific role of PUFAs and PUFAs-containing lipids in the pathophysiology of SCZ and MDD. It should be emphasized that the free PUFAs were not measured in the current study and were not reported as distinctive for SCZ and MDD in other research; therefore, we focus here on PUFAs-containing lipids. While there could be multiple explanations of the results found in our study, the decreased PUFAs-containing TG and PC levels we found in MDD may reflect their vulnerability to oxidative stress and inflammation [63,64]. Although oxidative stress and inflammation are not unique to MDD, and are also observed in SCZ [65,66], MDD is characterized by higher levels of inflammation-related factors, such as proinflammatory cytokines [67]. Therefore, future research may concentrate on the precise investigation of inflammatory markers in SCZ and MDD.

In addition to SCZ and MDD, our study included patients with bipolar (BPD), schizoaffective (AFF), and schizotypal (TYP) disorders. AFF and TYP are considered as schizophrenia-spectrum disorders, while BPD occupies an intermediate position between SCZ and MDD in the DSM-5, showing both manic (SCZ-like) and depressive symptoms [68,69]. We further tested whether the separation of SCZ and MDD patients based on the MDD-SCZ lipid panel could be generalized to the other psychotic and affective illnesses. Lipid profiles showed a clear separation of the disorders according to the affective-psychotic classification: SCZ and SCZ-like diseases clustered together along the first principal component (PC1), MDD formed a distinct group, and BPD samples aligned closer to MDD (Figure 5B,C). This arrangement mirrors the symptom-based categorization of these disorders, suggesting a general link between the blood levels of “discordant” lipids and symptom manifestations. Although we lacked quantitative symptom severity data, the observed lipid profile patterns qualitatively reflected the classic Kraepelinian dichotomy between psychotic and affective disorders [38]. Prior studies also linked lipid abundances with symptom severity in SCZ and MDD [21,28,62], supporting the idea that the abundance levels of particular lipids may indicate psychiatric patients’ condition.

To further support the idea that lipids can differentiate between mental disorders, we decided to build the classification algorithm for disease prediction. Recent works demonstrated the applicability of machine learning approaches such as logistic regression, extreme gradient boosting, and others, for psychiatric disorder classification. Specifically, such algorithms were applied for SCZ-CTR [17,20], MDD-CTR [21,36], BPD-CTR [36], BPD-MDD [31], BPD-SCZ [45], and SCZ-MDD [28,45] differentiation. In our research, we first constructed diseases-control models, specifically, SCZ-CTR and MDD-CTR, using lipid intensities. The obtained metrics were expectedly good and comparable with the previously reported results [17,36], confirming that there is a significant difference between SCZ or MDD and CTR. Then, we made similar model for SCZ and MDD patient classification. The datasets of both cohorts were utilized to train the model, which can make it more universal and unbiased. The model performance, with ROC AUC = 0.83, aligns with the results previously obtained for the SCZ-MDD comparison [28,45] and exceeds the performance of the model classifying MDD and BPD [31]. The model also demonstrated the higher ability to differentiate between SCZ and MDD in Cohort 1 than in another cohort (Table S24), probably due to a stronger MDD-SCZ difference effect in Cohort 1. Interestingly, the algorithm separated not only SCZ and MDD, which it was trained on, but also AFF and TYP, perfectly classifying it as SCZ, and BPD, classifying it as intermediate between SCZ and MDD (Figure S10). Of note, our study included a cross-dataset analysis for both SCZ and MDD, but BPD, AFF, and TYP were only represented in one of the two datasets, possibly limiting the reliability of our findings for these three disorders. While the features selected based on model performance did not fully match the MDD-SCZ lipid panel (Table S25), the possibility of separating the two disorders based on multivariate modelling demonstrated that lipidomic data can be a promising diagnostic tool for psychiatric disorders.

Our work, however, does not address the problem of systemic differences in medication between patient and control groups. Effects of antipsychotics and antidepressants on blood lipidomic content have been reported: for instance, the abundance levels of several PC, LPC, and CAR species were significantly altered after antipsychotic treatment in comparison to first-episode patients (FEP) [70]. Antidepressant intake similarly showed alterations of the abundance levels in blood plasma for a number of PC compounds [71]. As for TG, most of the study indicated that both antipsychotics and antidepressants lead to an increase of total TG, Chol, low-density lipoprotein cholesterol, and certain TG [22,72,73]; however, comprehensive information about the influence of medication on the lipid species is limited. Nonetheless, lack of substantial overlap between both concordant and discordant SCZ- and MDD-associated alterations and the lipids linked to pharmacological treatment suggest the independence of our conclusions from the medication effects. From another point of view, the medication factor, as well as dietary conditions, can be considered as limitations of the current study and may be incorporated as confounders in future research. Another limitation might be the low sample size of TYP, AFF, and BPD groups and their presence in only one of the cohorts, preventing the possibility to better estimate the ability of the lipid panel to differentiate psychotic and affective diseases.

## 5. Conclusions

To conclude, our study further supports the notion that blood plasma lipid levels can not only provide information about the presence of an unspecified psychiatric condition but also help to differentiate among affective and psychotic diseases. The experimental design, with the inclusion of samples from two independent cohorts, allowed us to estimate the reproducibility of the observed disorder-associated alterations and can be considered as one of the main advantages of the research. As the most important outcome, we identified a group of lipids, many of which contained PUFA residues, which separated both patient groups from CTR and from each other. They were altered in MDD relative to SCZ, suggesting the importance of PUFAs-containing lipids in pathophysiological processes during the disorders, for example, inflammation. In addition, we built a logistic regression model that separates not only SCZ and MDD but other psychotic and affective patient groups, such as schizotypal, schizoaffective, and bipolar disorders. These findings may be valuable in two key ways: first, they offer fundamental insights that could advance our understanding of the pathophysiological mechanisms underlying these diseases, which are currently unclear; second, they hold translational potential as a basis for clinical biomarker panels. Thus, while the accuracy of differentiating mental disorders requires improvement and consideration of clinical factors such as medication, blood lipidome profiling shows strong potential for advancing fundamental research and developing objective biochemical tools for psychiatric practice.

## Data Availability

Data needed to evaluate the results are present in the paper and/or the Supplementary Materials. Additional data, data processing codes, or other technical details can be provided upon reasonable request from the corresponding author.

## Supplementary Materials

The following supporting information can be downloaded at: https://www.mdpi.com/article/10.3390/biom15091296/s1.

## Abbreviations

The following abbreviations are used in the manuscript:

SCZ	Schizophrenia
MDD	Major depressive disorder
CTR	Control
BPD	Bipolar disorder
AFF	Schizoaffective disorder
TYP	Schizotypal disorder
BMI	Body mass index
DSM-5	Diagnostic and Statistical Manual of Mental Disorders
ICD-10	International Classification of Disease 10
CAR	Acylcarnitines
PC	Phosphatidylcholines
PC-O	Ether-linked phosphatidylcholines
PE	Phosphatidylethanolamines
PE-P	Plasmalogen phosphatidylethanolamines
LPC	Lysophosphatidylcholines
LPE	Lysophosphatidylethanolamines
Cer	Ceramides
SM	Sphingomyelins
Chol	Cholesterol
CE	Cholesterol Esters
DG	Diacylglycerols
TG	Triacylglycerols
PUFAs	Polyunsaturated fatty acids
DB	Double bonds
QC	Quality control
FC	Fold change
BH	Benjamini–Hochberg procedure
PCA	Principal component analysis
PLS-DA	Partial least square discriminant analysis
KS	Kolmogorov–Smirnov
KDE	Kernel density estimation
ROC AUC	Area under the receiver operating characteristic
