# Supplementary material for: Blood Plasma Lipid Alterations Differentiating Psychotic and Affective Disorder Patients
Source: Biomolecules. 2025 Sep 9;15(9):1296. doi: 10.3390/biom15091296 (PMC12467233; doi:10.3390/biom15091296)
Supplement: Supplementary file 1 [file biomolecules-15-01296-s001.zip › Supplementary Information and Figures.pdf]

Supplementary Information and Figures

Sample preparation

The required volumes of water and organic mixture were added to 20 µl of plasma. After 10 minutes of ultrasound exposure (50/60 Hz, Bandelin Electronic, Berlin, Germany) in ice bath, samples were vigorously shaken for 40 munities at 4 °C (Vortex Genie, Scientific Industries, New York, USA) and centrifuged afterwards for 10 minutes at 12700 rpm, 4 °C (Centrifuge 5427R, Eppendorf, Germany). Then 1000 µl of organic phase and 200 µl of water polar phase were manually taken from each sample. The organic phase was dried under reduced pressure. All the extracted samples were kept at -80 °C until the analysis and multiple thaw-freeze cycles were avoided.

Mass spectrometry DIA method

In this work flow injection mass spectrometry was used. Injection volume was set to 20 µl, the autosampler temperature maintained at 4 °C. The flow rate was modulated during the analytical run. The eluent flow rate was set to 0.8 ml/min in the intervals of 0-0.04 minutes and 2.01-3.0 minutes and maintained at 10 µl/min in the range of 0.04-2.01. Increased flow rate was used for sample introduction in the beginning and loop flushing at the end of the run. DIA method was set as follows: first, full scan range of interest (m/z 200-1050) was split into 8 narrow windows to avoid C-trap overload (spectral-stitching direct infusion mass spectrometry) with a resolution of 140,000 (FWFM at m/z 200). DIA event consisted of 1 Da-width windows within the range 200.5 -1050.5 m/z with a resolution of 17,500 (FWHM at m/z 200). All spectra were recorded in profile mode. Pierce LTQ Velos ESI Positive Ion Calibration Solution was used for external calibration in positive mode.

The source settings were established as follows for 10 µl/min flow: sheath gas, 15 a.u.; aux gas, 5 a.u.; sweep gas, 0 a.u.; spray voltage, 3.5 kV; capillary temperature, 250 °C; S-lens RF level, 50; aux heater temperature, 250 °C. For 800 µl/min, source parameters changed accordingly: sheath gas, 60 a.u.; aux gas, 20 a.u.; sweep gas, 4 a.u.; spray voltage, 2.5 kV; capillary temperature, 300 °C; S-lens RF level, 50; aux heater temperature, 300 °C. For MS1 spectra AGC was set at $5*10E^6$ and max IT time 100 ms. The splitting intervals for MS1 acquisition were chosen to maintain a consistent ion current across each m/z segment. The ion acquisition program was set as follows: 0.12-0.17 s: 200-652 Da, 0.17-0.22 s: 652 - 684 Da, 0.22-0.27 s: 684-716 Da, 0.27-0.32 s: 716-764 Da, 0.32-0.37 s: 764-812 Da, 0.37-0.42 s: 812-876 Da, 0.42-0.47 s: 876-908 Da, 0.47-0.52 s: 908-1051 Da. For MS2 acquisition collision energy was applied in step-wise manner (15-20-25), AGC target was set at $2*10E^5$, isolation window was 1.2 Da and fixed first mass equaled to m/z 80.

Lipid identification and data processing

Raw files were converted using PeakStrainer software keeping only MS1 information for biological samples and extraction blanks (time range of 0-32.5 sec) and MS1 and MS2 information for QC samples.

LipidXplorer was used for lipid annotation with the following parameters: selection window was set at 1 Da; time range was from 0.01 to 1000 s; MS1 range was 200-1050 m/z, MS/MS range was 80-1000 m/z. Resolution for MS1 was set at 140000, while MS/MS resolution was set at 17500 FMHW; tolerance was 5 ppm for MS1 and 20 ppm for MS/MS; threshold was chosen 0 for MS1 and 20000 for MS/MS (abs); resolution gradient was set at -100 res/(m/z) for MS and -90 for MS/MS. The rest parameters were set to 0. Lipid identification strategy was based on precursor high resolution MS1 information and MS2 fragmentation data using predefined MFQL files for LipidXplorer software.

Data handling included the following steps: first, duplicated (isomeric) features were removed; then, the lipids having missing values (NaN) in more than 10 % of samples were removed from analysis. The intensities were log_2-tranformed. Extraction blanks (empty samples undergoing all the steps of extraction and measurement) were used for filtration of non-biological contaminating features. Lipid species having at least 1.5 higher intensity in the samples than in blank were left. The NaN values were filled by minimum values calculated for each lipid. Batch correction was done for each lipid by subtracting the median QC for the current batch from all the samples included in this batch. At the last step highly variable lipids were filtered using QC samples. The variability was estimated by calculating the standard deviation (SD) for each species in QC. Typically, the lipids with SD 0-0.3 were left for further analysis. The datasets of Cohort 1 and Cohort 2 were measured separately and the intensities were aligned using long term reference (LTR) standard plasma samples.

MDD-SCZ classification model

Before the model construction the intensities were normalized by subtracting the mean of each lipid from samples and dividing by its standard deviation. Different values of the hyperparameter C (regularization strength parameter) were tested (specifically: 0.001, 0.01, 0.1, 1, 10, 100). The best value was chosen to be equal 1 using grid search technique (GridSeacrhCV function of sklearn Python package).

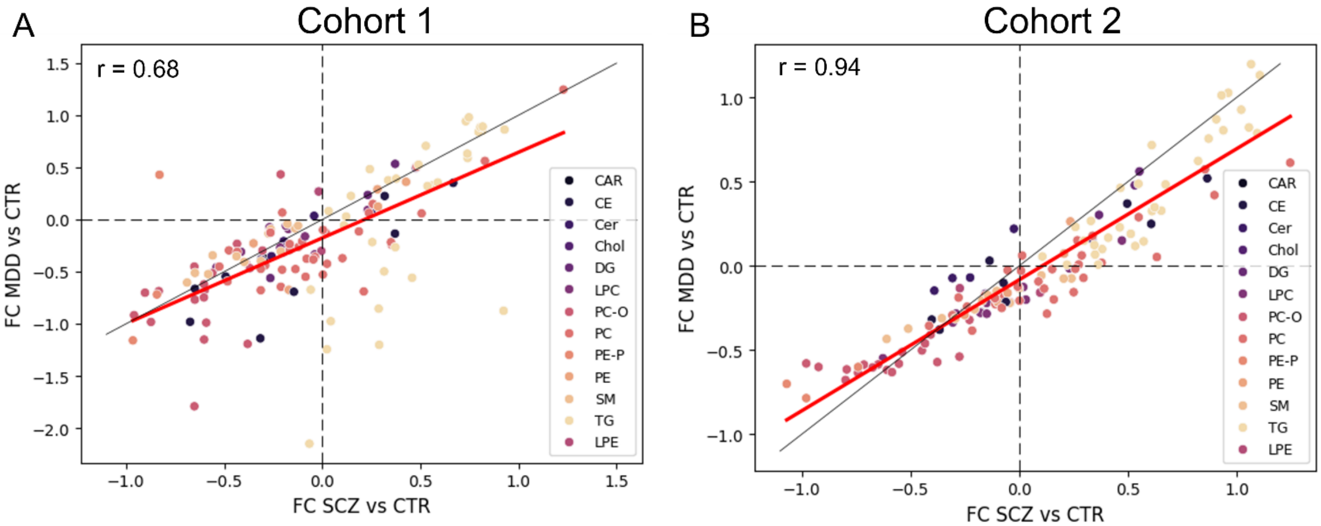

Figure S1. **(A)** Correlation of log2 fold changes (FC) in lipid abundances for SCZ vs CTR and MDD vs CTR of Cohort 1. r stands for Pearson correlation coefficient. **(B)** Same for Cohort 2.

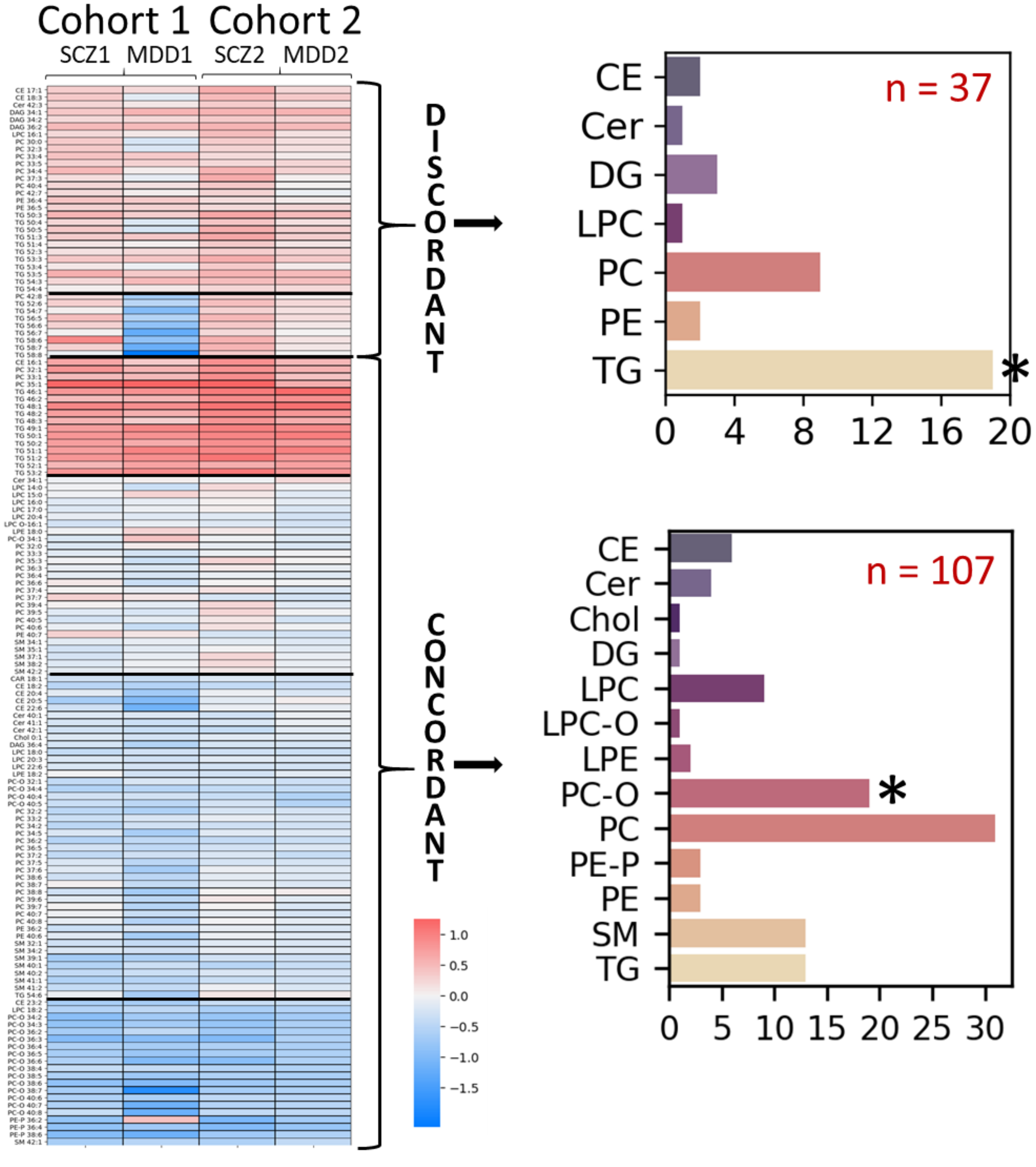

Figure S2. Heat map for k-means clustering of the disorder-against control FC (144 lipids changed at least in one cohort in total) for each cohort. Each cell depicts the value of FC SCZ-CTR or MDD-CTR for cohort 1 (number 1) or cohort 2 (number 2). Vertical brackets combine the discordant or concordant clusters of lipids showing different or similar behavior of SCZ and MDD vs CTR, respectively. Barplots represent distribution of lipids by classes, black asterisk shows the significance of hypergeometric enrichment test ($p < 0.05$).

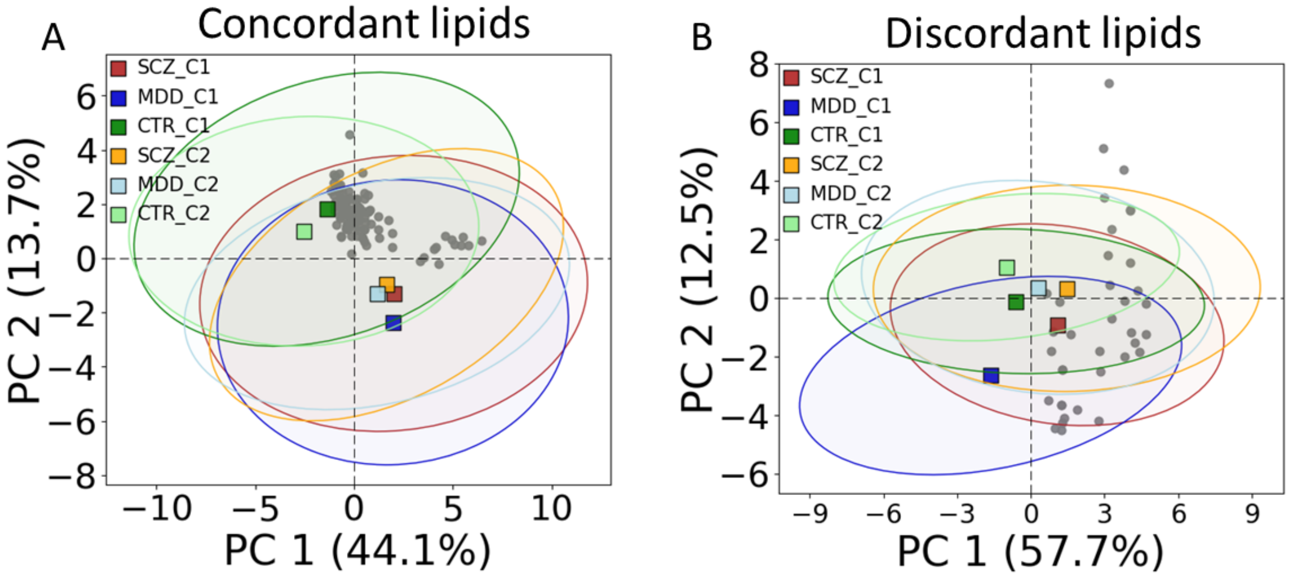

Figure S3. Principal Component Analysis (PCA) biplot performed on all samples from both cohorts using: **(A)** lipids from the concordant clusters; **(B)** lipids from discordant clusters; The borders of the ellipses depict the area inside which the points of that group are found with 95 % confidence. The squares inside each ellipse correspond to the mean values of PC1 and PC2 components of all points in that group (mass center). At x-axis the first principal component (PC 1) is plotted, while at y-axis the second principal component (PC 2) is plotted with the percentage of variation explained by this component in the brackets. The gray points belong to the lipids, the position is determined according to the loading score of that lipid for PC1 and PC2. For better visualization, the loading scores were multiplied by scaling factor = 18.

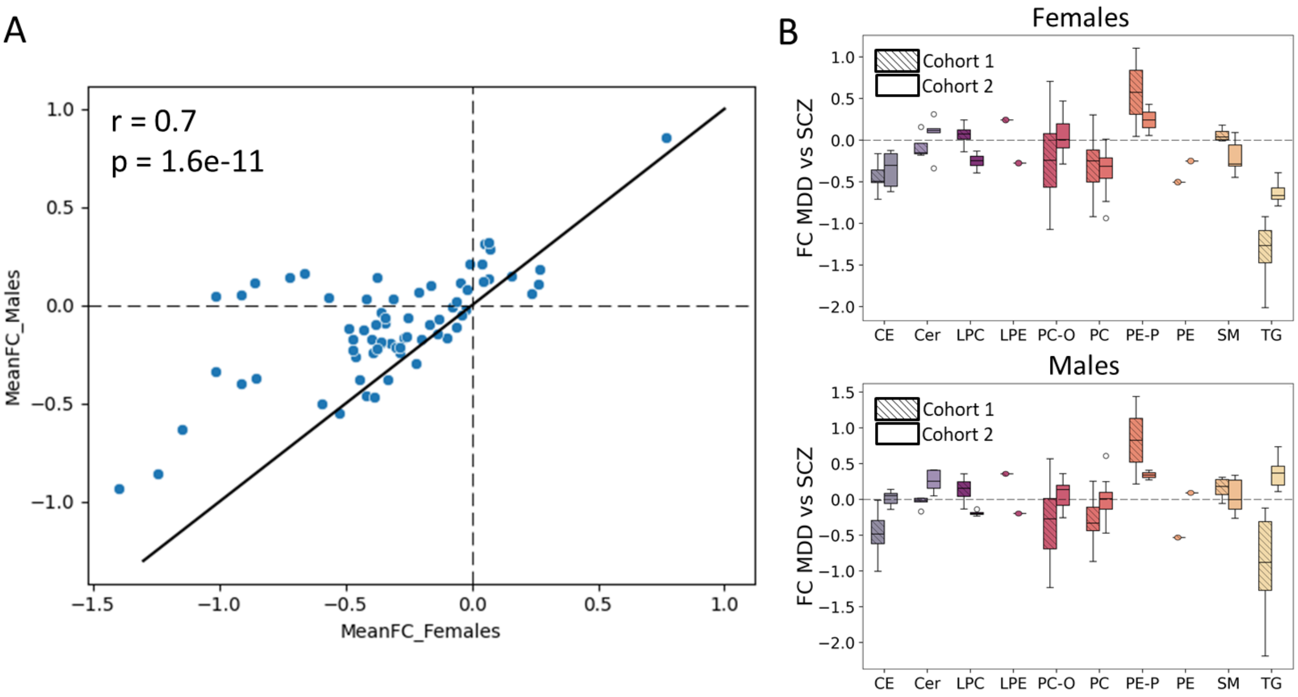

Figure S4. **(A)** Correlation of log2 fold changes (FC) in lipid abundances for MDD vs SCZ between males and females separately averaged for both cohorts (71 lipids common for both cohorts and sex). r stands for Pearson correlation coefficient. **(B)** boxplots showing distribution of these lipids by classes for both cohorts separately for males and females.

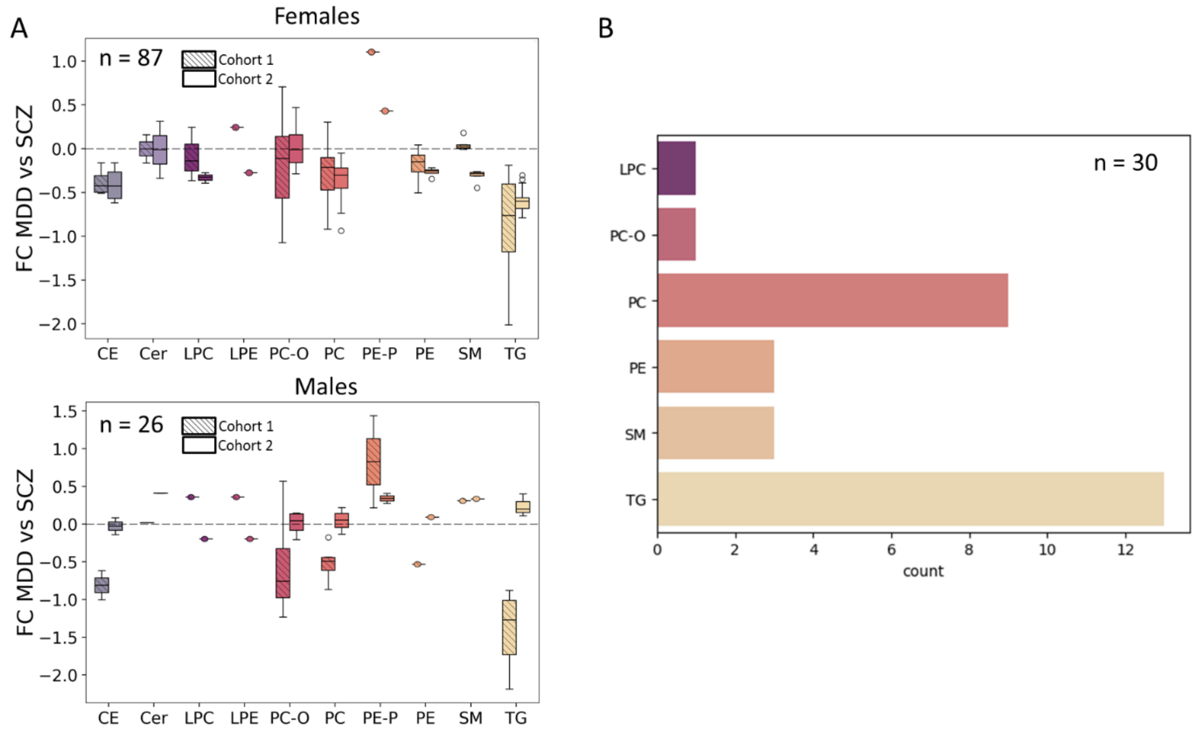

Figure S5. **(A)** boxplots showing the distribution of significantly altered lipids in females (top) and males (bottom); **(B)** Count of lipids potentially specific for females distributed by classes.

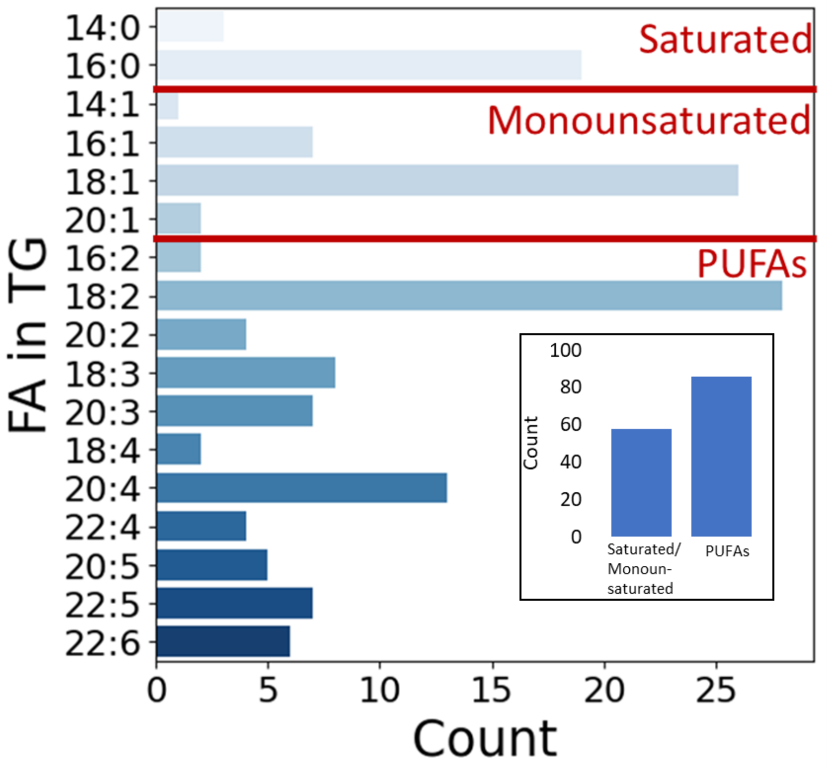

Figure S6. Distribution of fatty acid (FA) residues of all detected isomers TG among 20 “discordant” lipids. The insert barplot shows the number of FA residues: saturated/monounsaturated (left bar) and PUFAs (right bar).

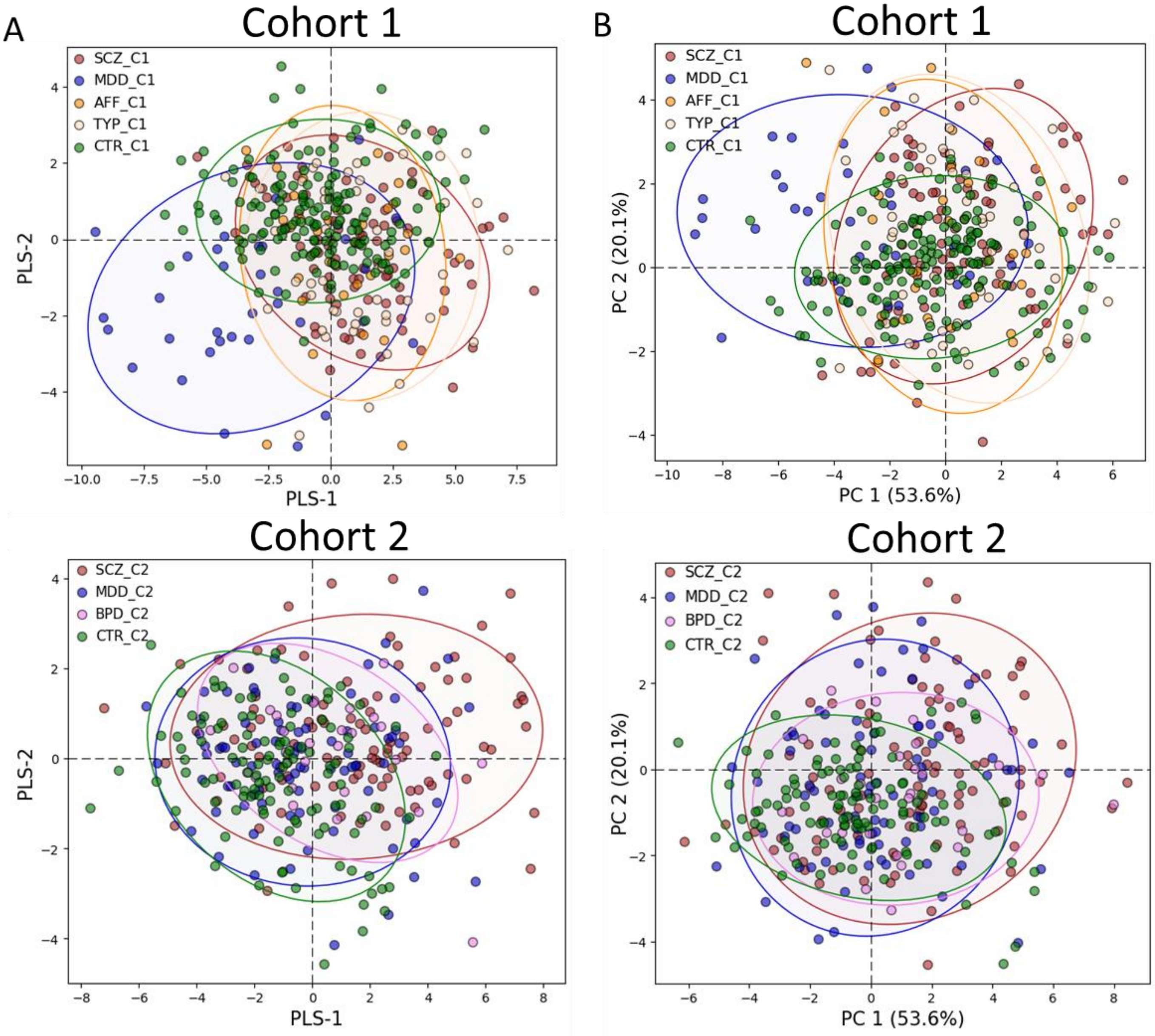

Figure S7. (A) Partial Least Square Discriminant Analysis (PLS-DA) for cohorts 1 and 2. **(B)** Principal Component Analysis (PCA) for cohorts 1 and 2.

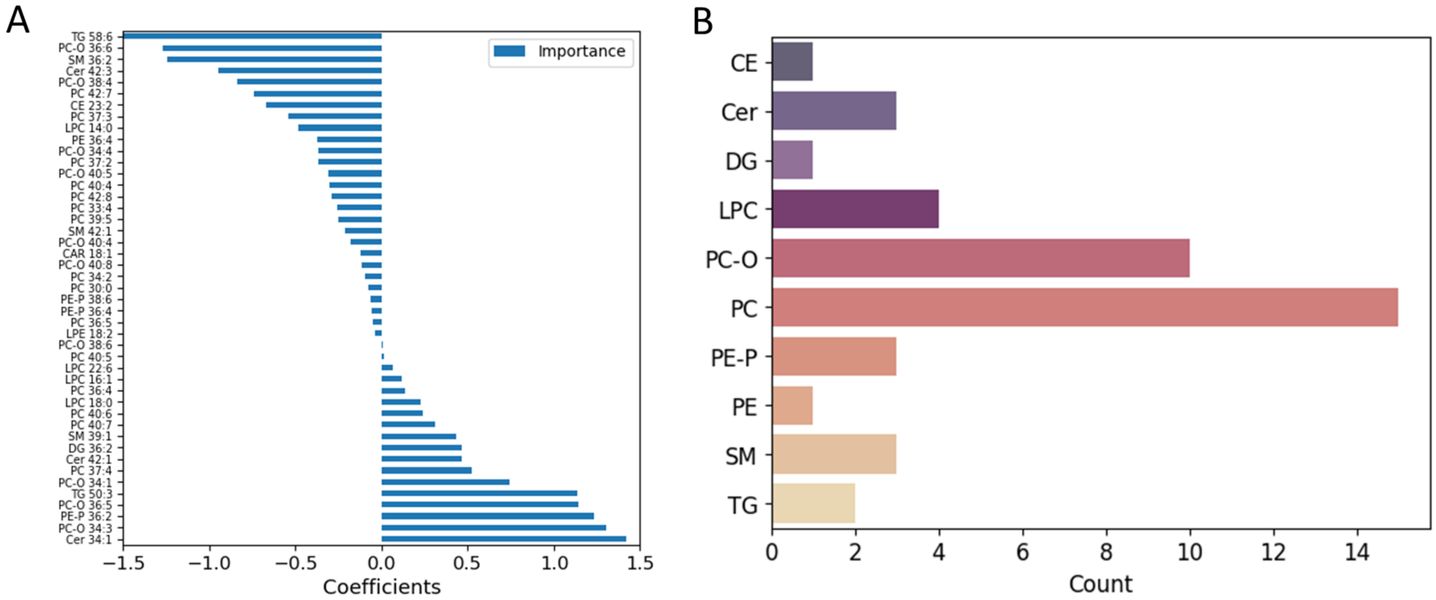

Figure S8. **(A)** The lipid species with non-zero coefficients for the constructed MDD-SCZ logistic regression model. **(B)** The distribution of these lipid species by classes.

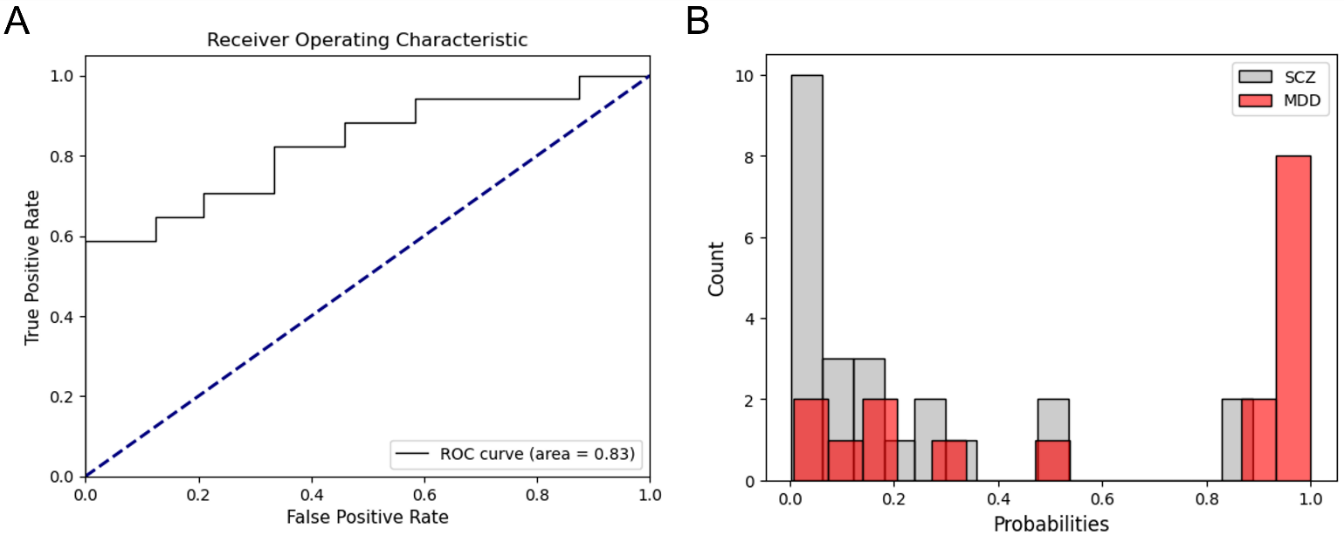

Figure S9. **(A)** ROC AUC for the MDD-SCZ logistic regression model. **(B)** The distribution of the probability scores obtained for test dataset of MDD-SCZ logistic regression model.

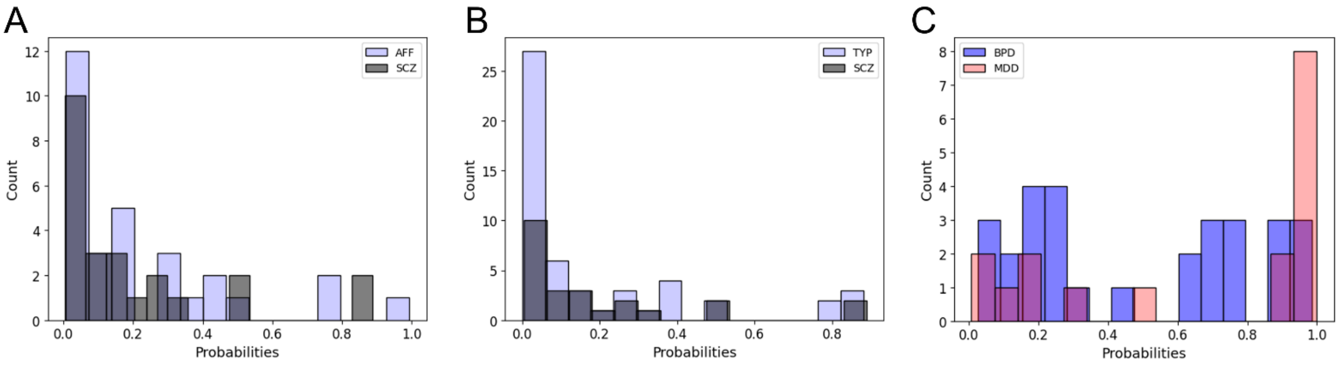

Figure S10. The distribution of the probability scores obtained from the constructed logistic regression model for samples of dataset containing: **(A)** AFF; **(B)** TYP, and **(C)** BPD, diagnostic groups of logistic regression model.
