# Supplementary material for: Blood Plasma Lipid Alterations Differentiating Psychotic and Affective Disorder Patients
Source: Biomolecules. 2025 Sep 9;15(9):1296. doi: 10.3390/biom15091296 (PMC12467233; doi:10.3390/biom15091296)
Supplement: Supplementary file 1 [file biomolecules-15-01296-s001.zip › Supplementary Tables.pdf]

Supplementary Tables

Table S1. Demographic data for Cohort 1 and Cohort 2.

	Group	Total	Ratio woman/men	Age	BMI
**Cohort 1**	Schizophrenia (F20)	85	0.9	33±1.2	24±0.5
	Schizotypal (F21)	52	0.7	31.3±2.1	23.7±0.9
	Schizoaffective (F25)	30	0.6	34.1±2.8	23.6±1
	Depression (F32,33)	35	1.2	33.2±3.3	22.5±1.3
	Control	160	1	33.7±1.3	24±0.3
**Cohort 2**	Schizophrenia (F20)	100	1.3	39.7±1.6	23.9±0.9
	Bipolar (F31)	29	0.7	34.1±3.7	25.7±0.9
	Depression (F32,33)	85	1.8	40.8±2.4	23.2±0.7
	Control	112	1.5	32.3±1.5	24.4±0.8

Table S2. Number of samples for training and test sets used for logistic regression model.

	Training set				Test set			
	**SCZ**	**MDD**	**ratio SCZ/MDD**	**ratio Cohort1/ Cohort 2**	**SCZ**	**MDD**	**ratio SCZ/MDD**	**ratio Cohort1/Cohort 2**
**Cohort 1**	40	25	1.6	0.65	10	7	1.4	0.71
**Cohort 2**	62	38	1.6		14	10	1.4	

Table S3: Log2-transformed FC in lipid abundances for SCZ against CTR for Cohort1. "color_condition" column depicts the significance of the lipid difference (two-sample two-sided *t*-test): "red" for BH-corrected $p < 0.05$, "black" for BH-corrected $p > 0.05$.

Lipid	logFC	BH-corrected P.value	color_condition
CAR 18:1	-0.29448	1.2437E-05	red
CE 16:1	0.66764	2.74222E-11	red
CE 17:1	0.318393	1.6352E-05	red
CE 18:2	-0.49192	9.33244E-18	red
CE 18:3	0.370987	0.002732148	red
CE 20:5	-0.67314	5.2079E-06	red
CE 22:6	-0.31515	0.004202251	red
CE 23:2	-0.6487	3.6124E-19	red
Cer 40:1	-0.19634	0.001198859	red
Cer 41:1	-0.25829	5.79041E-05	red
Cer 42:1	-0.31103	1.45364E-07	red
Cer 42:3	0.255832	0.000149126	red
Chol 0:1	-0.19565	3.42528E-06	red
DG 34:1	0.371853	0.001471668	red

DG 34:2	0.232215	0.018316777	red
DG 36:2	0.47768	4.21583E-06	red
DG 36:4	-0.26423	0.015538853	red
LPC 16:0	-0.17223	0.000383839	red
LPC 16:1	0.231925	0.00100654	red
LPC 17:0	-0.18486	0.009805111	red
LPC 18:0	-0.41353	4.26994E-09	red
LPC 18:2	-0.53887	1.03308E-08	red
LPC 20:3	-0.20032	0.002591735	red
LPC 22:6	-0.21407	0.007588466	red
LPC O-16:1	-0.26873	3.25394E-05	red
PC 30:0	0.353953	0.000916301	red
PC 32:0	-0.20348	0.000148519	red
PC 32:1	0.829308	1.66337E-13	red
PC 32:2	-0.48247	1.14851E-16	red
PC 32:3	0.350594	0.000265258	red
PC 33:1	0.482531	3.74676E-06	red
PC 33:2	-0.35015	2.52309E-07	red
PC 33:3	-0.16538	0.045861001	red
PC 33:4	0.427354	4.58806E-07	red
PC 33:5	0.281781	0.00119893	red
PC 34:2	-0.4753	1.04034E-21	red
PC 34:4	0.506614	3.35385E-09	red
PC 34:5	-0.2106	0.006075199	red
PC 35:1	1.229071	4.50753E-15	red
PC 36:2	-0.5241	3.6124E-19	red
PC 36:4	-0.12003	0.023955109	red
PC 36:5	-0.29028	2.68902E-10	red
PC 37:2	-0.43326	1.78903E-09	red
PC 37:5	-0.20426	0.000148519	red
PC 37:6	-0.17023	0.007588466	red
PC 37:7	0.284184	0.000157907	red
PC 38:6	-0.31263	2.67644E-06	red
PC 38:8	-0.31293	0.00372442	red
PC 40:4	0.258226	0.003137018	red
PC 42:7	0.238095	0.001984617	red
PC 42:8	0.211916	0.007858642	red
PC-O 32:1	-0.43388	1.87224E-13	red
PC-O 34:1	-0.21168	0.000635472	red
PC-O 34:2	-0.90296	2.56398E-27	red
PC-O 34:3	-0.83256	7.60725E-24	red
PC-O 34:4	-0.5602	5.62422E-10	red
PC-O 36:2	-0.65154	2.69367E-19	red

PC-O 36:3	-0.95864	3.94996E-13	red
PC-O 36:4	-0.59854	2.81357E-21	red
PC-O 36:5	-0.64898	1.39422E-19	red
PC-O 36:6	-0.59079	1.04394E-09	red
PC-O 38:4	-0.38894	2.10914E-08	red
PC-O 38:5	-0.60264	6.84993E-20	red
PC-O 38:6	-0.8725	9.92532E-27	red
PC-O 38:7	-0.6515	1.85421E-23	red
PC-O 40:4	-0.3044	0.000418663	red
PC-O 40:5	-0.36821	7.09962E-07	red
PC-O 40:6	-0.60227	2.07661E-18	red
PC-O 40:7	-0.60175	4.04709E-20	red
PC-O 40:8	-0.37993	9.45984E-13	red
PE 36:2	-0.35015	2.52309E-07	red
PE 36:4	0.427354	4.58806E-07	red
PE 36:5	0.281781	0.00119893	red
PE 40:6	-0.17023	0.007588466	red
PE 40:7	0.284184	0.000157907	red
PE-P 36:2	-0.82925	1.34699E-18	red
PE-P 36:4	-0.84231	2.08068E-20	red
PE-P 38:6	-0.96467	2.30911E-37	red
SM 32:1	-0.31214	1.09506E-05	red
SM 34:1	-0.13822	0.004240261	red
SM 34:2	-0.21092	0.000635472	red
SM 35:1	-0.27306	6.28235E-05	red
SM 37:1	-0.26246	0.00321229	red
SM 38:2	-0.17681	0.004202251	red
SM 39:1	-0.68851	6.74186E-16	red
SM 40:1	-0.55823	2.4766E-16	red
SM 40:2	-0.4364	4.26211E-12	red
SM 41:1	-0.58095	3.1197E-16	red
SM 41:2	-0.39732	8.0743E-08	red
SM 42:1	-0.64836	8.56995E-17	red
SM 42:2	-0.1227	0.045317892	red
TG 46:1	0.815719	0.004240261	red
TG 48:1	0.929193	5.36295E-05	red
TG 48:2	0.741275	0.000196605	red
TG 48:3	0.562266	0.00453397	red
TG 49:1	0.732015	0.000351127	red
TG 50:1	0.797109	4.51531E-06	red
TG 50:2	0.739769	1.52998E-07	red
TG 50:3	0.536755	4.28025E-05	red
TG 50:5	0.372654	0.017688288	red

TG 51:1	0.747567	0.001946234	red
TG 51:2	0.806301	8.23626E-07	red
TG 51:3	0.336414	0.010796162	red
TG 52:1	0.528263	0.000196605	red
TG 52:6	0.315753	0.028664392	red
TG 53:2	0.817796	6.50378E-07	red
TG 53:3	0.376978	0.003350574	red
TG 53:5	0.588909	2.80282E-06	red
TG 54:3	0.242604	0.037571951	red
TG 56:5	0.458283	2.67644E-06	red
TG 56:6	0.287936	0.001345954	red
TG 58:6	0.924397	1.21686E-12	red
TG 58:7	0.290626	0.016023569	red
Cer 42:2	0.072586	0.209884004	black
PC 36:6	0.103038	0.209884004	black
PC 37:3	0.187199	0.174627026	black
PC 37:4	0.003962	0.960604883	black
PC 38:7	0.024853	0.690240737	black
PC 39:4	0.017719	0.826163128	black
PC 39:7	0.003637	0.960604883	black
TG 46:2	0.490994	0.051322545	black
TG 50:4	0.254925	0.093694007	black
TG 51:4	0.12187	0.342804299	black
TG 52:3	0.146617	0.169403944	black
TG 52:5	0.187957	0.150742274	black
TG 53:4	0.111471	0.459436971	black
TG 54:4	0.032657	0.788067428	black
TG 54:5	0.00608	0.960604883	black
TG 54:7	0.043817	0.777235074	black
TG 56:7	0.023977	0.827583505	black
CE 16:0	-0.06685	0.155711972	black
CE 20:4	-0.14413	0.054458701	black
Cer 34:1	-0.04109	0.322855671	black
DG 36:3	-0.0448	0.704867081	black
LPC 14:0	-0.03802	0.72578542	black
LPC 15:0	-0.01787	0.777235074	black
LPC 18:1	-0.1066	0.157706545	black
LPC 20:4	-0.08751	0.176533385	black
LPE 18:0	-0.01787	0.777235074	black
LPE 18:2	-0.00434	0.961138091	black
PC 34:3	-0.02935	0.715473201	black
PC 35:3	-0.03677	0.668722399	black
PC 35:5	-0.02655	0.72578542	black

PC 36:3	-0.10201	0.079097718	black
PC 39:5	-0.16599	0.056763404	black
PC 39:6	-0.13635	0.106540541	black
PC 40:5	-0.26358	0.173708888	black
PC 40:6	-0.07938	0.37014854	black
PC 40:7	-0.04508	0.498297649	black
PC 40:8	-0.08472	0.159248713	black
SM 36:1	-0.09664	0.152741708	black
SM 36:2	-0.13442	0.060461642	black
TG 52:4	-0.08207	0.510976464	black
TG 54:6	-0.05847	0.66111755	black
TG 58:8	-0.0652	0.650953827	black

Table S4: Log2-transformed FC in lipid abundances for SCZ against CTR for Cohort2. "color_condition" column depicts the significance of the lipid difference (two-sample two-sided t-test): "red" for BH-corrected $p < 0.05$, "black" for BH-corrected $p > 0.05$.

**Lipid**	**logFC**	**BH-corrected P.value**	**color_condition**
CE 16:1	0.864486	3.89E-15	red
CE 17:1	0.606689	5.13E-12	red
CE 18:3	0.49635	1.01E-05	red
Cer 42:3	0.335012	3.68E-08	red
DG 34:1	0.552471	5.09E-07	red
DG 34:2	0.366787	0.000312	red
DG 36:2	0.531056	1.77E-06	red
LPC 14:0	0.230224	0.007801	red
LPC 16:1	0.472032	1.69E-09	red
PC 30:0	0.337285	0.001278	red
PC 32:1	0.854203	3.7E-12	red
PC 32:3	0.267137	0.003146	red
PC 33:1	0.897423	1.99E-12	red
PC 33:4	0.206132	0.00469	red
PC 33:5	0.234965	0.003057	red
PC 34:4	0.549084	5.42E-09	red
PC 35:1	1.245806	5.55E-15	red
PC 35:3	0.27348	0.005129	red
PC 37:3	0.632637	2.11E-07	red
PC 39:4	0.250263	0.001859	red
PC 39:5	0.257636	0.000349	red
PC 40:4	0.379255	1.26E-05	red
PC 42:7	0.295378	1.21E-05	red
PC 42:8	0.287241	9.66E-06	red

PE 36:4	0.206132	0.00469	red
PE 36:5	0.234965	0.003057	red
SM 37:1	0.240346	0.001617	red
SM 38:2	0.204385	0.000579	red
TG 46:1	1.066044	3.11E-05	red
TG 46:2	0.906053	4.59E-05	red
TG 48:1	1.106342	1.08E-06	red
TG 48:2	0.938254	1.72E-06	red
TG 48:3	0.822384	1.82E-05	red
TG 49:1	1.020611	4.48E-07	red
TG 50:1	0.961228	5.24E-08	red
TG 50:2	0.870598	1.07E-08	red
TG 50:3	0.672751	3.94E-06	red
TG 50:4	0.40919	0.007312	red
TG 50:5	0.61005	3.39E-05	red
TG 51:1	0.930201	6.01E-06	red
TG 51:2	1.09392	3.8E-10	red
TG 51:3	0.653046	5.49E-06	red
TG 51:4	0.345253	0.006915	red
TG 52:1	0.609232	2.02E-05	red
TG 52:3	0.329465	0.003763	red
TG 52:6	0.462288	0.001151	red
TG 53:2	1.056151	6.05E-10	red
TG 53:3	0.622328	5.49E-06	red
TG 53:4	0.348695	0.010451	red
TG 53:5	0.548256	1.26E-05	red
TG 54:3	0.464924	0.000106	red
TG 54:4	0.257974	0.026408	red
TG 54:7	0.297821	0.049907	red
TG 56:5	0.51277	1.66E-05	red
TG 56:6	0.381024	0.000655	red
TG 58:6	0.571996	2.33E-05	red
TG 58:7	0.53301	0.000166	red
TG 58:8	0.362985	0.014829	red
CE 18:2	-0.40225	2.17E-19	red
CE 23:2	-0.36627	7.29E-14	red
Cer 40:1	-0.30701	2.45E-08	red
Cer 41:1	-0.22581	2.9E-05	red
Cer 42:1	-0.3904	7.99E-12	red
Chol 0:1	-0.15605	2.82E-05	red
DG 36:4	-0.24769	0.023755	red
LPC 18:0	-0.30543	1.08E-06	red
LPC 18:2	-0.62685	1.65E-12	red

LPC 20:3	-0.14984	0.04549	red
LPC 22:6	-0.29029	0.000233	red
LPC O-16:1	-0.17854	0.00477	red
LPE 18:2	-0.27326	0.003763	red
PC-O 32:1	-0.22895	2.27E-05	red
PC-O 34:2	-0.79889	1.4E-24	red
PC-O 34:3	-0.79328	2.34E-26	red
PC-O 34:4	-0.40699	2.22E-13	red
PC-O 36:2	-0.7141	1.08E-24	red
PC-O 36:3	-0.97925	6.07E-16	red
PC-O 36:4	-0.6082	8.6E-22	red
PC-O 36:5	-0.64881	1.71E-23	red
PC-O 36:6	-0.92284	1.68E-29	red
PC-O 38:4	-0.55313	3.28E-14	red
PC-O 38:5	-0.58527	1.86E-19	red
PC-O 38:6	-0.74207	2.34E-26	red
PC-O 38:7	-0.67398	2.34E-26	red
PC-O 40:4	-0.37682	3.44E-07	red
PC-O 40:5	-0.27504	1.27E-05	red
PC-O 40:6	-0.44725	5.49E-13	red
PC-O 40:7	-0.53852	5.22E-17	red
PC-O 40:8	-0.43298	1.18E-17	red
PC 32:0	-0.10941	0.022155	red
PC 32:2	-0.24152	0.000286	red
PC 34:2	-0.30744	1.52E-13	red
PC 36:2	-0.41476	8.6E-17	red
PC 36:5	-0.279	1.01E-13	red
PC 37:2	-0.21673	0.001294	red
PC 37:5	-0.23988	5.7E-08	red
PC 37:7	-0.25114	4.51E-05	red
PC 38:7	-0.11153	0.032577	red
PE-P 36:2	-1.06916	7.83E-26	red
PE-P 36:4	-0.9794	2.09E-29	red
PE-P 38:6	-0.74081	3.4E-24	red
PE 40:7	-0.25114	4.51E-05	red
SM 32:1	-0.17036	0.006607	red
SM 34:1	-0.14408	0.00068	red
SM 39:1	-0.2937	9.89E-06	red
SM 40:1	-0.51033	2.8E-18	red
SM 40:2	-0.20559	8.89E-05	red
SM 41:1	-0.35583	1.14E-09	red
SM 42:1	-0.60956	1.13E-17	red
Cer 42:2	0.058745	0.274779	black

DG 36:3	0.028847	0.794531	black
LPC 15:0	0.096014	0.120222	black
LPC 16:0	0.018536	0.705655	black
LPC 17:0	0.030491	0.671428	black
LPC 18:1	0.002279	0.984453	black
LPE 18:0	0.096014	0.120222	black
PC 34:3	0.075629	0.261676	black
PC 36:3	0.088483	0.124416	black
PC 36:6	0.008204	0.92366	black
PC 37:4	0.15871	0.054679	black
PC 38:8	0.01307	0.925483	black
PC 39:6	0.114626	0.190331	black
PC 39:7	0.001189	0.984453	black
PC 40:5	0.127358	0.34002	black
PC 40:6	0.151467	0.107868	black
SM 36:1	0.07227	0.266317	black
SM 36:2	0.082359	0.236673	black
SM 42:2	0.101575	0.063788	black
TG 52:4	0.061369	0.633872	black
TG 52:5	0.257199	0.056838	black
TG 54:5	0.153165	0.212196	black
TG 54:6	0.106159	0.426963	black
TG 56:7	0.219433	0.07552	black
CAR 18:1	-0.17677	0.053403	black
CE 16:0	-0.01974	0.655313	black
CE 20:4	-0.06132	0.370692	black
CE 20:5	-0.13638	0.430604	black
CE 22:6	-0.07221	0.483767	black
Cer 34:1	-0.02455	0.635181	black
LPC 20:4	-0.14944	0.060353	black
PC-O 34:1	-0.04063	0.537477	black
PC 33:2	-0.099	0.133213	black
PC 33:3	-0.05343	0.483767	black
PC 34:5	-0.15663	0.113772	black
PC 35:5	-0.07432	0.14453	black
PC 36:4	-0.07257	0.174131	black
PC 37:6	-0.0536	0.391499	black
PC 38:6	-0.12011	0.060895	black
PC 40:7	-0.08071	0.190331	black
PC 40:8	-0.00686	0.925483	black
PE 36:2	-0.099	0.133213	black
PE 40:6	-0.0536	0.391499	black
SM 34:2	-0.10382	0.058571	black

SM 35:1	-0.00126	0.984453	black
SM 41:2	-0.04432	0.483767	black

Table S5: Log2-transformed FC in lipid abundances for MDD against CTR for Cohort1. "color_condition" column depicts the significance of the lipid difference (two-sample two-sided t-test): "red" for BH-corrected $p < 0.05$, "black" for BH-corrected $p > 0.05$.

Lipid	logFC	BH-corrected P.value	color_condition
CE 16:1	0.351791	0.007822	red
DG 34:1	0.533793	0.000343	red
DG 34:2	0.236249	0.047068	red
DG 36:2	0.497621	0.000556	red
LPC 15:0	0.2698	0.006894	red
LPE 18:0	0.2698	0.006894	red
PC-O 34:1	0.433689	1.56E-06	red
PC 32:1	0.562174	0.001053	red
PC 33:1	0.506926	0.000481	red
PC 33:4	0.361764	0.000593	red
PC 33:5	0.290524	0.014046	red
PC 35:1	1.246695	1.97E-09	red
PE-P 36:2	0.42961	0.022738	red
PE 36:4	0.361764	0.000593	red
PE 36:5	0.290524	0.014046	red
TG 46:1	0.850896	0.023041	red
TG 48:1	0.863127	0.00326	red
TG 48:2	0.589922	0.020648	red
TG 49:1	0.943021	0.000672	red
TG 50:1	0.840041	0.000413	red
TG 50:2	0.631885	0.001146	red
TG 51:1	0.979893	0.005218	red
TG 51:2	0.886268	0.000147	red
TG 51:3	0.377278	0.047877	red
TG 52:1	0.706951	0.000995	red
TG 53:2	0.891935	0.000185	red
TG 53:3	0.392509	0.0367	red
TG 54:3	0.483569	0.008978	red
CAR 18:1	-0.3702	0.003331	red
CE 18:2	-0.54559	1.56E-08	red
CE 20:4	-0.6924	5.57E-07	red
CE 20:5	-0.98011	2.05E-05	red
CE 22:6	-1.1386	3.46E-07	red
CE 23:2	-0.66387	5.34E-05	red
Cer 40:1	-0.20655	0.012838	red

Cer 41:1	-0.35135	0.000147	red
Cer 42:1	-0.37862	4.34E-06	red
Chol 0:1	-0.26701	5.03E-05	red
DG 36:4	-0.56083	0.000366	red
LPC 14:0	-0.3336	0.003462	red
LPC 18:0	-0.30885	0.001888	red
LPC 18:2	-0.45783	0.00011	red
LPC 20:3	-0.3137	0.000931	red
LPC 22:6	-0.26891	0.013686	red
LPE 18:2	-0.30096	0.018359	red
PC-O 32:1	-0.2321	0.005802	red
PC-O 34:2	-0.70112	1.97E-09	red
PC-O 34:3	-0.68573	4.73E-07	red
PC-O 34:4	-0.51243	3.56E-06	red
PC-O 36:2	-0.45054	1.27E-05	red
PC-O 36:3	-0.91613	1.91E-06	red
PC-O 36:4	-0.67619	2.24E-12	red
PC-O 36:5	-0.7681	3.29E-12	red
PC-O 36:6	-0.98848	5.03E-11	red
PC-O 38:4	-0.41513	0.000163	red
PC-O 38:5	-0.74656	7.08E-12	red
PC-O 38:6	-0.98166	2.24E-12	red
PC-O 38:7	-1.79032	2.95E-11	red
PC-O 40:4	-0.29664	0.014913	red
PC-O 40:5	-0.46546	2.52E-06	red
PC-O 40:6	-0.62045	1.3E-09	red
PC-O 40:7	-1.1502	3.45E-10	red
PC-O 40:8	-1.19235	1.99E-10	red
PC 32:2	-0.5927	1.2E-10	red
PC 33:2	-0.22778	0.023041	red
PC 34:2	-0.2952	2.83E-06	red
PC 34:5	-0.64434	6.02E-07	red
PC 35:3	-0.3572	0.013686	red
PC 36:2	-0.45072	2.23E-08	red
PC 36:3	-0.20998	0.013811	red
PC 36:4	-0.26186	0.000318	red
PC 36:5	-0.439	1.47E-10	red
PC 36:6	-0.37039	0.000559	red
PC 37:2	-0.30945	0.000663	red
PC 37:5	-0.48041	3.15E-07	red
PC 37:6	-0.67372	3.46E-07	red
PC 38:6	-0.47274	2.78E-06	red
PC 38:7	-0.4239	7.27E-07	red

PC 38:8	-0.67729	5.19E-05	red
PC 39:6	-0.47469	0.001232	red
PC 39:7	-0.52754	2.07E-05	red
PC 40:6	-0.37556	0.004246	red
PC 40:7	-0.38249	5.19E-05	red
PC 40:8	-0.54858	1.18E-06	red
PC 42:8	-0.68909	5.02E-06	red
PE-P 36:4	-0.71827	7.96E-05	red
PE-P 38:6	-1.15842	1.01E-14	red
PE 36:2	-0.22778	0.023041	red
PE 40:6	-0.67372	3.46E-07	red
SM 32:1	-0.39836	0.00018	red
SM 34:2	-0.30518	0.000453	red
SM 39:1	-0.60352	2.78E-06	red
SM 40:1	-0.34312	4.27E-05	red
SM 40:2	-0.35193	0.000102	red
SM 41:1	-0.52319	1.33E-07	red
SM 41:2	-0.40582	0.000274	red
SM 42:1	-0.51146	1.94E-06	red
TG 52:6	-0.49717	0.025665	red
TG 54:6	-0.6723	0.001822	red
TG 54:7	-0.9746	0.000117	red
TG 56:5	-0.55802	0.001256	red
TG 56:6	-0.85401	7.7E-06	red
TG 56:7	-1.24356	3.46E-07	red
TG 58:6	-0.87444	0.000283	red
TG 58:7	-1.20119	2.78E-06	red
TG 58:8	-2.14908	8.96E-09	red
CE 17:1	0.22593	0.060013	black
Cer 34:1	0.036049	0.610156	black
Cer 42:3	0.09979	0.321625	black
LPC 16:1	0.091932	0.355068	black
PC 32:0	0.067296	0.411675	black
PC 34:4	0.059127	0.645229	black
PC 35:5	0.028736	0.787288	black
PC 37:7	0.124919	0.337985	black
PC 40:4	0.15049	0.156749	black
PC 42:7	0.063912	0.551369	black
PE 40:7	0.124919	0.337985	black
TG 46:2	0.526043	0.112884	black
TG 48:3	0.32768	0.220349	black
TG 50:3	0.321575	0.085213	black
TG 51:4	0.029412	0.863891	black

TG 52:3	0.22687	0.144429	black
TG 53:5	0.355807	0.065989	black
TG 54:4	0.081568	0.634407	black
CE 16:0	-0.07103	0.326641	black
CE 18:3	-0.13484	0.32191	black
Cer 42:2	-0.08044	0.318271	black
DG 36:3	-0.1486	0.30082	black
LPC 16:0	-0.09026	0.275193	black
LPC 17:0	-0.05959	0.573975	black
LPC 18:1	-0.03681	0.711461	black
LPC 20:4	-0.16138	0.075036	black
LPC O-16:1	-0.07358	0.523624	black
PC 30:0	-0.24177	0.08366	black
PC 32:3	-0.21653	0.075036	black
PC 33:3	-0.29162	0.050386	black
PC 34:3	-0.01153	0.880166	black
PC 37:3	-0.11331	0.551369	black
PC 37:4	-0.09911	0.332056	black
PC 39:4	-0.12549	0.282574	black
PC 39:5	-0.23467	0.05814	black
PC 40:5	-0.12484	0.516951	black
SM 34:1	-0.0849	0.230709	black
SM 35:1	-0.13393	0.217137	black
SM 36:1	-0.03634	0.691772	black
SM 36:2	-0.06743	0.526645	black
SM 37:1	-0.09329	0.450657	black
SM 38:2	-0.04107	0.678502	black
SM 42:2	-0.059	0.503657	black
TG 50:4	-0.21597	0.340101	black
TG 50:5	-0.26512	0.260085	black
TG 52:4	-0.14197	0.415306	black
TG 52:5	-0.36885	0.075036	black
TG 53:4	-0.05534	0.777118	black
TG 54:5	-0.2787	0.130719	black

Table S6: Log2-transformed FC in lipid abundances for MDD against CTR for Cohort2. "color_condition" column depicts the significance of the lipid difference (two-sample two-sided t-test): "red" for BH-corrected $p < 0.05$, "black" for BH-corrected $p > 0.05$.

**Lipid**	**logFC**	**BH-corrected P.value**	**color_condition**
CE 16:1	0.519535	1.61E-06	red
CE 17:1	0.248727	0.002027	red

CE 18:3	0.369287	0.000952	red
Cer 34:1	0.219942	4.64E-06	red
Cer 42:3	0.157069	0.010509	red
DG 34:1	0.56028	1.17E-05	red
DG 34:2	0.302107	0.005893	red
DG 36:2	0.478374	6.69E-05	red
PC 32:1	0.572567	7.57E-06	red
PC 33:1	0.419718	0.000211	red
PC 33:5	0.263126	0.006251	red
PC 34:4	0.288641	0.004741	red
PC 35:1	0.611939	0.000408	red
PE 36:5	0.263126	0.006251	red
TG 46:1	1.197118	3.53E-05	red
TG 46:2	0.86994	0.000584	red
TG 48:1	1.131514	7.57E-06	red
TG 48:2	0.804469	0.000197	red
TG 48:3	0.622609	0.002632	red
TG 49:1	0.926599	2.65E-05	red
TG 50:1	1.027236	1.36E-07	red
TG 50:2	0.755209	3.45E-06	red
TG 50:3	0.482894	0.001261	red
TG 50:5	0.324702	0.041239	red
TG 51:1	1.012132	1.84E-05	red
TG 51:2	0.787325	1.26E-05	red
TG 51:3	0.327526	0.024842	red
TG 52:1	0.716561	9.27E-06	red
TG 52:3	0.266565	0.022063	red
TG 53:2	0.821351	7.73E-06	red
TG 53:3	0.34753	0.014151	red
TG 53:5	0.487078	0.000362	red
TG 54:3	0.462369	0.000386	red
CAR 18:1	-0.27554	0.004463	red
CE 18:2	-0.31949	2.27E-12	red
CE 20:4	-0.21495	0.005893	red
CE 23:2	-0.3793	9.56E-09	red
Cer 42:1	-0.14743	0.009427	red
Chol 0:1	-0.16623	8.4E-05	red
DG 36:4	-0.26419	0.018599	red
LPC 15:0	-0.15872	0.013041	red
LPC 16:0	-0.12605	0.027753	red
LPC 17:0	-0.20115	0.006166	red
LPC 18:0	-0.33656	3.45E-06	red
LPC 18:2	-0.549	2.25E-07	red

LPC 20:3	-0.22045	0.006414	red
LPC 20:4	-0.28464	0.000768	red
LPC 22:6	-0.28376	0.000814	red
LPC O-16:1	-0.27913	0.000236	red
LPE 18:0	-0.15872	0.013041	red
PC-O 32:1	-0.3209	2.8E-08	red
PC-O 34:1	-0.21685	0.002244	red
PC-O 34:2	-0.6781	3E-15	red
PC-O 34:3	-0.61506	7.56E-18	red
PC-O 34:4	-0.40826	1.92E-11	red
PC-O 36:2	-0.58696	7.8E-15	red
PC-O 36:3	-0.57942	8.11E-08	red
PC-O 36:4	-0.61827	2.44E-19	red
PC-O 36:5	-0.58593	2.2E-18	red
PC-O 36:6	-0.60061	6.52E-14	red
PC-O 38:4	-0.58093	7.62E-12	red
PC-O 38:5	-0.63132	5.01E-18	red
PC-O 38:6	-0.63461	1.06E-17	red
PC-O 38:7	-0.60325	2.11E-17	red
PC-O 40:4	-0.57173	2.43E-07	red
PC-O 40:5	-0.53932	8.28E-10	red
PC-O 40:6	-0.50274	2.61E-16	red
PC-O 40:7	-0.51251	2.11E-15	red
PC-O 40:8	-0.4611	3.06E-13	red
PC 32:0	-0.22814	1.37E-05	red
PC 32:2	-0.25275	0.000817	red
PC 33:2	-0.21513	0.002664	red
PC 34:2	-0.29264	6.89E-10	red
PC 36:2	-0.35613	5.24E-11	red
PC 36:4	-0.18483	0.000755	red
PC 36:5	-0.18854	1.1E-05	red
PC 37:2	-0.38472	5.5E-08	red
PC 37:4	-0.19972	0.010509	red
PC 37:5	-0.24245	4.22E-06	red
PC 37:6	-0.16106	0.011699	red
PC 37:7	-0.29181	2.65E-05	red
PC 38:6	-0.17904	0.004418	red
PC 38:7	-0.19206	0.001292	red
PC 39:4	-0.1564	0.018923	red
PC 39:7	-0.20684	0.003001	red
PC 40:5	-0.28458	0.037818	red
PC 40:7	-0.25022	1.34E-05	red
PC 40:8	-0.12318	0.047072	red

PE-P 36:2	-0.70081	4.29E-12	red
PE-P 36:4	-0.78549	9.75E-20	red
PE-P 38:6	-0.60079	3.69E-15	red
PE 36:2	-0.21513	0.002664	red
PE 40:6	-0.16106	0.011699	red
PE 40:7	-0.29181	2.65E-05	red
SM 32:1	-0.20689	0.001261	red
SM 34:1	-0.17478	5.79E-05	red
SM 34:2	-0.14932	0.004027	red
SM 35:1	-0.13438	0.047072	red
SM 39:1	-0.31312	7.57E-06	red
SM 40:1	-0.37316	5.89E-12	red
SM 40:2	-0.25839	7.59E-07	red
SM 41:1	-0.31157	7E-07	red
SM 41:2	-0.22069	0.000585	red
SM 42:1	-0.43429	3.34E-10	red
CE 20:5	0.030188	0.90024	black
Cer 42:2	0.068392	0.216225	black
DG 36:3	0.005492	0.960593	black
LPC 16:1	0.150261	0.081059	black
PC 30:0	0.178157	0.119019	black
PC 32:3	0.150994	0.139584	black
PC 33:4	0.072586	0.388695	black
PC 35:3	0.007586	0.956931	black
PC 37:3	0.052298	0.736145	black
PC 38:8	0.061251	0.705906	black
PC 40:4	0.017621	0.850742	black
PC 42:8	0.061789	0.345832	black
PE 36:4	0.072586	0.388695	black
TG 50:4	0.166704	0.31782	black
TG 51:4	0.123029	0.378039	black
TG 52:4	0.008725	0.956931	black
TG 52:5	0.076855	0.596097	black
TG 52:6	0.228384	0.145815	black
TG 53:4	0.067037	0.673893	black
TG 54:4	0.230917	0.055738	black
TG 54:5	0.096194	0.452412	black
TG 54:6	0.055271	0.705906	black
TG 54:7	0.148997	0.366315	black
TG 56:5	0.189708	0.103633	black
TG 56:6	0.09922	0.378039	black
TG 56:7	0.006528	0.960593	black
TG 58:6	0.14504	0.29668	black

TG 58:7	0.117657	0.409369	black
TG 58:8	0.006669	0.962437	black
CE 16:0	-0.07153	0.143457	black
CE 22:6	-0.1017	0.35285	black
Cer 40:1	-0.07286	0.187904	black
Cer 41:1	-0.06981	0.245577	black
LPC 14:0	-0.01484	0.90024	black
LPC 18:1	-0.1116	0.233615	black
LPE 18:2	-0.13296	0.257752	black
PC 33:3	-0.02647	0.798832	black
PC 34:3	-0.05268	0.468166	black
PC 34:5	-0.17599	0.104148	black
PC 35:5	-0.07838	0.216225	black
PC 36:3	-0.09333	0.125169	black
PC 36:6	-0.02949	0.705906	black
PC 39:5	-0.0178	0.837346	black
PC 39:6	-0.13527	0.104148	black
PC 40:6	-0.01965	0.849725	black
PC 42:7	-0.07158	0.388695	black
SM 36:1	-0.05461	0.388695	black
SM 36:2	-0.08089	0.233615	black
SM 37:1	-0.07484	0.392208	black
SM 38:2	-0.0759	0.257752	black
SM 42:2	-0.05706	0.282811	black

Table S7. p-values and BH-adjusted p-values of one-sample Wilcox test (FC SCZ vs CTR of lipids within a class is compared to 0) for 1 and 2 cohorts by classes. Significant p-values ($p < 0.05$) are marked in red.

	**Cohort 1**		**Cohort 2**	
**Class**	**p_value**	**p_adjusted**	**p_value**	**p_adjusted**
CE	3.13E-01	5.36E-01	1.00E+00	1.00E+00
Cer	6.25E-01	8.33E-01	3.75E-01	5.00E-01
DG	2.50E-01	5.00E-01	3.75E-01	5.00E-01
LPC	4.38E-01	6.56E-01	3.13E-01	5.00E-01
PC	1.45E-01	4.34E-01	2.75E-01	5.00E-01
PC-O	7.63E-06	4.58E-05	7.63E-06	4.58E-05
PE	1.00E+00	1.00E+00	2.50E-01	5.00E-01
PE-P	2.50E-01	5.00E-01	2.50E-01	5.00E-01
SM	9.77E-02	3.91E-01	3.91E-03	1.56E-02
TG	4.77E-07	5.72E-06	4.77E-07	5.72E-06

Table S8. p-values and BH-adjusted p-values of one-sample Wilcox test (FC MDD vs CTR of lipids within a class is compared to 0) for 1 and 2 cohorts by classes. Significant p-values ($p < 0.05$) are marked in red.

	Cohort 1		Cohort 2	
**Class**	**p_value**	**p_adjusted**	**p_value**	**p_adjusted**
CE	2.50E-01	5.42E-01	8.75E-01	1.00E+00
DG	8.75E-01	1.00E+00	3.75E-01	6.96E-01
LPC	1.88E-01	4.88E-01	6.25E-02	2.03E-01
PC	7.99E-02	2.60E-01	2.75E-01	5.97E-01
PC-O	2.67E-05	3.47E-04	3.81E-06	4.96E-05
PE	7.50E-01	1.00E+00	1.00E+00	1.00E+00
PE-P	5.00E-01	9.29E-01	2.50E-01	5.97E-01
SM	7.81E-03	3.39E-02	7.81E-03	3.39E-02
TG	4.88E-04	3.17E-03	4.88E-04	3.17E-03

Table S9: Results of k-means clustering analysis of averaged log2-transformed fold changes (FC) between SCZ or MDD vs CTR for each lipid for both cohorts. In "cluster" column there are numbers of clusters the lipids are belonging to according to k-means.

Lipid	SCZ Cohort1	MDD Cohort1	SCZ Cohort2	MDD Cohort2	cluster
CE 17:1	0.31839279	0.225930247	0.60668869	0.248727011	1
CE 18:3	0.37098681	-0.13483518	0.49635015	0.369286695	1
Cer 42:3	0.2558316	0.099790255	0.33501166	0.157068761	1
DG 34:1	0.37185289	0.533792763	0.55247072	0.560280392	1
DG 34:2	0.2322146	0.236248802	0.36678733	0.30210676	1
DG 36:2	0.47768009	0.497621221	0.53105584	0.478373907	1
LPC 16:1	0.23192451	0.091932267	0.4720321	0.150261284	1
PC 30:0	0.35395299	-0.24177479	0.33728487	0.178157136	1
PC 32:3	0.35059404	-0.21653193	0.26713661	0.150994136	1
PC 33:4	0.42735417	0.361763975	0.20613151	0.072585746	1
PC 33:5	0.28178144	0.290523581	0.2349648	0.263126074	1
PC 34:4	0.50661445	0.059126674	0.54908423	0.288640801	1
PC 37:3	0.18719925	-0.11330545	0.6326365	0.052298372	1
PC 40:4	0.25822579	0.150490495	0.37925452	0.017620719	1
PC 42:7	0.23809526	0.063911953	0.29537784	-0.07157503	1
PE 36:4	0.42735417	0.361763975	0.20613151	0.072585746	1
PE 36:5	0.28178144	0.290523581	0.2349648	0.263126074	1
TG 50:3	0.53675492	0.32157494	0.67275132	0.482893936	1
TG 50:4	0.2549246	-0.2159698	0.40918977	0.166704107	1
TG 50:5	0.3726545	-0.26512269	0.61004961	0.324701537	1
TG 51:3	0.3364141	0.377277764	0.65304556	0.327525583	1
TG 51:4	0.12187026	0.029412087	0.34525301	0.123028735	1
TG 52:3	0.1466173	0.226870498	0.3294648	0.266564899	1

TG 53:3	0.37697803	0.392508914	0.62232829	0.347529994	1
TG 53:4	0.11147102	-0.05533824	0.34869522	0.067036731	1
TG 53:5	0.58890857	0.355807269	0.5482558	0.487078264	1
TG 54:3	0.24260407	0.483568637	0.46492413	0.462368592	1
TG 54:4	0.03265679	0.081567795	0.25797439	0.230916781	1
PC 42:8	0.21191641	-0.68908741	0.28724114	0.061788624	2
TG 52:6	0.31575294	-0.49716984	0.46228805	0.228383747	2
TG 54:7	0.04381749	-0.9745956	0.29782078	0.148997076	2
TG 56:5	0.45828324	-0.55801888	0.51276967	0.189708475	2
TG 56:6	0.2879357	-0.85401078	0.3810237	0.099219569	2
TG 56:7	0.0239772	-1.24355978	0.21943305	0.006528391	2
TG 58:6	0.92439673	-0.87443664	0.57199553	0.14504023	2
TG 58:7	0.29062578	-1.20119455	0.53301049	0.11765719	2
TG 58:8	-0.065195	-2.14907519	0.36298483	0.006669315	2
CE 16:1	0.66764044	0.351791296	0.86448616	0.519534728	3
PC 32:1	0.82930795	0.562174339	0.85420324	0.572567049	3
PC 33:1	0.48253105	0.506926379	0.8974227	0.419717583	3
PC 35:1	1.22907103	1.246695219	1.24580561	0.611939051	3
TG 46:1	0.81571919	0.850896301	1.06604416	1.197117726	3
TG 46:2	0.4909937	0.526042628	0.90605328	0.8699401	3
TG 48:1	0.92919335	0.863127375	1.1063421	1.131513551	3
TG 48:2	0.74127524	0.589922126	0.93825411	0.804469337	3
TG 48:3	0.56226625	0.327679908	0.82238421	0.622608841	3
TG 49:1	0.73201515	0.943021363	1.02061057	0.926599051	3
TG 50:1	0.79710907	0.840040977	0.96122769	1.027236369	3
TG 50:2	0.73976896	0.631885054	0.87059756	0.755208835	3
TG 51:1	0.74756723	0.97989288	0.93020053	1.012132354	3
TG 51:2	0.80630085	0.886267837	1.09391993	0.787324818	3
TG 52:1	0.52826281	0.706950839	0.60923153	0.716561057	3
TG 53:2	0.81779604	0.891935108	1.05615109	0.821351418	3
Cer 34:1	-0.0410864	0.036049213	-0.02454974	0.219942268	4
LPC 14:0	-0.0380246	-0.33360001	0.23022402	-0.01484283	4
LPC 15:0	-0.0178667	0.269799749	0.09601438	-0.15871761	4
LPC 16:0	-0.1722275	-0.09026168	0.01853578	-0.12605122	4
LPC 17:0	-0.184857	-0.05958926	0.03049059	-0.2011463	4
LPC 20:4	-0.0875103	-0.16138213	-0.14944182	-0.28464168	4
LPC O-16:1	-0.2687304	-0.07358256	-0.17853672	-0.27913061	4
LPE 18:0	-0.0178667	0.269799749	0.09601438	-0.15871761	4
PC-O 34:1	-0.2116786	0.433688576	-0.04062583	-0.21684968	4
PC 32:0	-0.2034778	0.067295864	-0.10941476	-0.22813859	4
PC 33:3	-0.1653789	-0.29162184	-0.05342559	-0.02647128	4
PC 35:3	-0.0367706	-0.35720407	0.27347989	0.007585733	4
PC 36:3	-0.1020084	-0.20997917	0.08848332	-0.09333061	4

PC 36:4	-0.1200293	-0.26185785	-0.07257144	-0.1848297	4
PC 36:6	0.10303779	-0.37039229	0.0082044	-0.02949463	4
PC 37:4	0.00396231	-0.09910675	0.15870985	-0.19971804	4
PC 37:7	0.28418354	0.124919072	-0.25113837	-0.29181322	4
PC 39:4	0.01771947	-0.12548546	0.25026266	-0.15639948	4
PC 39:5	-0.1659923	-0.2346715	0.25763633	-0.01779748	4
PC 40:5	-0.2635811	-0.12484191	0.12735776	-0.28457989	4
PC 40:6	-0.0793775	-0.37555781	0.15146702	-0.01965363	4
PE 40:7	0.28418354	0.124919072	-0.25113837	-0.29181322	4
SM 34:1	-0.1382204	-0.08489737	-0.14407666	-0.17477884	4
SM 35:1	-0.2730592	-0.13393123	-0.00126222	-0.13437734	4
SM 37:1	-0.2624581	-0.09328881	0.24034635	-0.07484421	4
SM 38:2	-0.1768113	-0.04106868	0.20438515	-0.07590386	4
SM 42:2	-0.1226972	-0.05899663	0.10157456	-0.05706281	4
CAR 18:1	-0.2944832	-0.37019845	-0.17677236	-0.27554115	5
CE 18:2	-0.4919165	-0.54559192	-0.40224941	-0.31948838	5
CE 20:4	-0.144131	-0.69239521	-0.06131763	-0.21495005	5
CE 20:5	-0.6731384	-0.98010818	-0.13638276	0.030187688	5
CE 22:6	-0.3151532	-1.13860463	-0.07220833	-0.10169872	5
Cer 40:1	-0.1963359	-0.20654891	-0.30701433	-0.07285798	5
Cer 41:1	-0.2582903	-0.3513526	-0.22580925	-0.06981442	5
Cer 42:1	-0.3110313	-0.37862266	-0.39039998	-0.14742842	5
Chol 0:1	-0.1956484	-0.26700657	-0.15604503	-0.1662302	5
DG 36:4	-0.2642252	-0.56082585	-0.24768592	-0.26418769	5
LPC 18:0	-0.413529	-0.30884855	-0.30543055	-0.33655627	5
LPC 20:3	-0.200324	-0.31369611	-0.14983789	-0.22044894	5
LPC 22:6	-0.214071	-0.26891403	-0.29028639	-0.28376111	5
LPE 18:2	-0.0043392	-0.30095764	-0.27326225	-0.13296499	5
PC-O 32:1	-0.4338826	-0.23210096	-0.22894707	-0.32089551	5
PC-O 34:4	-0.5601981	-0.51242886	-0.40698987	-0.40826059	5
PC-O 40:4	-0.3043999	-0.29663853	-0.3768198	-0.57172561	5
PC-O 40:5	-0.3682088	-0.46546278	-0.2750389	-0.53932372	5
PC 32:2	-0.4824746	-0.59270164	-0.24151636	-0.25275292	5
PC 33:2	-0.3501513	-0.22777651	-0.09900415	-0.21512727	5
PC 34:2	-0.475304	-0.29519978	-0.3074371	-0.2926436	5
PC 34:5	-0.2106019	-0.64433854	-0.15663255	-0.17599491	5
PC 36:2	-0.5241047	-0.45072213	-0.41475703	-0.35612782	5
PC 36:5	-0.2902761	-0.43900224	-0.27900385	-0.18853903	5
PC 37:2	-0.433256	-0.30944712	-0.21673401	-0.38472442	5
PC 37:5	-0.2042631	-0.48040698	-0.23987905	-0.2424471	5
PC 37:6	-0.1702255	-0.67372291	-0.05359707	-0.16106417	5
PC 38:6	-0.3126347	-0.47274304	-0.12011238	-0.17903921	5
PC 38:7	0.02485285	-0.42390472	-0.11153493	-0.19206247	5

PC 38:8	-0.3129297	-0.67728738	0.0130703	0.061251039	5
PC 39:6	-0.136355	-0.4746923	0.11462636	-0.13527014	5
PC 39:7	0.00363669	-0.52754088	0.0011894	-0.20683754	5
PC 40:7	-0.0450751	-0.38248575	-0.08071099	-0.25022464	5
PC 40:8	-0.0847215	-0.54858341	-0.00686465	-0.12317936	5
PE 36:2	-0.3501513	-0.22777651	-0.09900415	-0.21512727	5
PE 40:6	-0.1702255	-0.67372291	-0.05359707	-0.16106417	5
SM 32:1	-0.3121434	-0.39836282	-0.17036006	-0.20688506	5
SM 34:2	-0.2109242	-0.30518169	-0.10381985	-0.14932101	5
SM 39:1	-0.6885115	-0.60352419	-0.29370268	-0.31312257	5
SM 40:1	-0.5582306	-0.34311639	-0.51032699	-0.37315936	5
SM 40:2	-0.4363956	-0.35193373	-0.20559082	-0.25838525	5
SM 41:1	-0.5809485	-0.52319314	-0.35582603	-0.31157456	5
SM 41:2	-0.3973191	-0.40581941	-0.0443183	-0.22068747	5
TG 54:6	-0.0584742	-0.67229534	0.10615882	0.055271419	5
CE 23:2	-0.6486953	-0.66386882	-0.3662686	-0.37929969	6
LPC 18:2	-0.5388696	-0.45782823	-0.62684951	-0.54900173	6
PC-O 34:2	-0.9029598	-0.70111553	-0.79888516	-0.67810171	6
PC-O 34:3	-0.8325604	-0.68572738	-0.79327514	-0.61505999	6
PC-O 36:2	-0.6515395	-0.45053929	-0.71410156	-0.58695598	6
PC-O 36:3	-0.9586365	-0.91613478	-0.97924777	-0.579416	6
PC-O 36:4	-0.5985376	-0.67619213	-0.60820123	-0.61827152	6
PC-O 36:5	-0.6489819	-0.76810416	-0.64880752	-0.58593495	6
PC-O 36:6	-0.5907874	-0.98847614	-0.92284074	-0.60061237	6
PC-O 38:4	-0.3889403	-0.4151311	-0.55313053	-0.58093087	6
PC-O 38:5	-0.6026375	-0.74656045	-0.58527017	-0.63132388	6
PC-O 38:6	-0.872498	-0.98166296	-0.74207482	-0.63461176	6
PC-O 38:7	-0.6514988	-1.79032173	-0.67397616	-0.60324511	6
PC-O 40:6	-0.6022745	-0.6204473	-0.44725478	-0.50273889	6
PC-O 40:7	-0.601747	-1.1502002	-0.53852438	-0.51250914	6
PC-O 40:8	-0.3799272	-1.19235176	-0.43298271	-0.46110051	6
PE-P 36:2	-0.8292526	0.429609924	-1.06916165	-0.70080803	6
PE-P 36:4	-0.8423062	-0.71826702	-0.97939987	-0.7854936	6
PE-P 38:6	-0.9646695	-1.15842074	-0.74081077	-0.60078581	6
SM 42:1	-0.6483619	-0.51146421	-0.60955937	-0.43429435	6

Table S10. p-values and BH-adjusted p-values of Mann Whitney U test for the significance of the differences in FC of SCZ vs CTR and MDD vs CTR of each cluster in each cohort.

**Cluster**	**Cohort 1**		**Cohort 2**		**Classification**
	**p_value**	**p_adjusted**	**p_value**	**p_adjusted**	
1	0.007475	0.014949	0.000074	0.000223	Discordant
2	0.000206	0.000618	0.000287	0.000574	Discordant

3	0.388716	0.466459	0.013734	0.020601	Concordant
4	0.5	0.5	0.000046	0.000223	Concordant
5	0.000153	0.000618	0.136227	0.136227	Concordant
6	0.114347	0.171521	0.023396	0.028075	Concordant

Table S11. p-values and BH-adjusted p-values of hypergeometric test for enrichment of a particular lipid class in the discordant clusters among lipids of this class in all clusters.

**Class**	**p-value**	**p_adjusted**
CE	0.656902	1
Cer	0.778988	1
DG	0.052241	0.365684
LPC	0.954216	1
PC	0.77321	1
PE	0.382025	1
TG	0.000003	0.000048

Table S12. Contingency table for Fisher's exact test of DB number in TG of discordant vs concordant lipid clusters. P.value = 0.000297.

	**Number of DB**	
	>=4	<4
Discordant	14	5
Concordant	1	12

Table S13. p-values and BH-adjusted p-values of hypergeometric test for enrichment of a particular lipid class in the concordant clusters among lipids of this class in all clusters.

**Class**	**p-value**	**p_adjusted**
CAR	0.743056	0.945707
CE	0.663034	0.945707
Cer	0.617975	0.945707
Chol	0.743056	0.945707
DG	0.996155	1
LPC	0.218641	0.945707
LPC O-	0.743056	0.945707
LPE	0.550796	0.945707
PC-O	0.002243	0.031402
PC	0.37579	0.945707
PE-P	0.407279	0.945707
PE	0.892795	1
SM	0.017217	0.120516
TG	1	1

Table S14: Log2-transformed FC in lipid abundances for MDD against SCZ for Cohort1. "color_condition" column depicts the significance of the lipid difference (two-sample two-sided t-test): "red" for BH-corrected p < 0.05, "black" for BH-corrected p > 0.05.

Lipid	logFC	BH-corrected P.value	color_condition
CE 18:3	-0.50582	0.005923	red
CE 20:4	-0.54826	0.000184	red
CE 22:6	-0.82345	0.000306	red
LPC 15:0	0.287666	0.01691	red
LPE 18:0	0.287666	0.01691	red
PC 30:0	-0.59573	0.000468	red
PC 32:0	0.270774	0.005923	red
PC 32:3	-0.56713	0.000184	red
PC 34:2	0.180104	0.012793	red
PC 34:4	-0.44749	0.005213	red
PC 34:5	-0.43374	0.001268	red
PC 36:5	-0.14873	0.037981	red
PC 36:6	-0.47343	0.000238	red
PC 37:5	-0.27614	0.005213	red
PC 37:6	-0.5035	0.000184	red
PC 38:7	-0.44876	1.41E-06	red
PC 38:8	-0.36436	0.037981	red
PC 39:7	-0.53118	0.000122	red
PC 40:7	-0.33741	0.001502	red
PC 40:8	-0.46386	0.000102	red
PC 42:8	-0.901	2.01E-07	red
PC-O 34:1	0.645367	1.88E-09	red
PC-O 36:6	-0.39769	0.00767	red
PC-O 38:7	-1.13882	1.18E-06	red
PC-O 40:7	-0.54845	0.00071	red
PC-O 40:8	-0.81242	1.65E-06	red
PE 40:6	-0.5035	0.000184	red
PE-P 36:2	1.258862	1.65E-07	red
SM 40:1	0.215114	0.035906	red
TG 50:5	-0.63778	0.022259	red
TG 52:5	-0.55681	0.025829	red
TG 52:6	-0.81292	0.002119	red
TG 54:6	-0.61382	0.012523	red
TG 54:7	-1.01841	0.000238	red
TG 56:5	-1.0163	6.61E-07	red
TG 56:6	-1.14195	1.65E-07	red
TG 56:7	-1.26754	7.37E-07	red
TG 58:6	-1.79883	2.56E-09	red

TG 58:7	-1.49182	1.76E-07	red
TG 58:8	-2.08388	9.6E-08	red
Cer 34:1	0.077136	0.470956	black
DG 34:1	0.16194	0.476349	black
DG 34:2	0.004034	0.972381	black
DG 36:2	0.019941	0.955162	black
LPC 16:0	0.081966	0.491478	black
LPC 17:0	0.125268	0.444674	black
LPC 18:0	0.10468	0.486672	black
LPC 18:1	0.06979	0.66671	black
LPC 18:2	0.081041	0.66671	black
LPC O-16:1	0.195148	0.201938	black
PC-O 32:1	0.201782	0.056204	black
PC-O 34:2	0.201844	0.116339	black
PC-O 34:3	0.146833	0.406123	black
PC-O 34:4	0.047769	0.78858	black
PC-O 36:2	0.201	0.109628	black
PC-O 36:3	0.042502	0.922933	black
PC-O 40:4	0.007761	0.958667	black
PC 33:1	0.024395	0.946017	black
PC 33:2	0.122375	0.406123	black
PC 33:5	0.008742	0.955162	black
PC 34:3	0.017819	0.930589	black
PC 35:1	0.017624	0.955162	black
PC 35:5	0.055291	0.730656	black
PC 36:2	0.073383	0.492163	black
PC 37:2	0.123809	0.383387	black
PC 40:5	0.138739	0.683383	black
PE-P 36:4	0.124039	0.634624	black
PE 36:2	0.122375	0.406123	black
PE 36:5	0.008742	0.955162	black
SM 34:1	0.053323	0.637373	black
SM 35:1	0.139128	0.389359	black
SM 36:1	0.060299	0.678849	black
SM 36:2	0.066994	0.679038	black
SM 37:1	0.169169	0.383387	black
SM 38:2	0.135743	0.383387	black
SM 39:1	0.084987	0.650419	black
SM 40:2	0.084462	0.51652	black
SM 41:1	0.057755	0.66671	black
SM 42:1	0.136898	0.377308	black
SM 42:2	0.063701	0.650419	black
TG 46:1	0.035177	0.955162	black

TG 46:2	0.035049	0.955162	black
TG 49:1	0.211006	0.630506	black
TG 50:1	0.042932	0.930589	black
TG 51:1	0.232326	0.66671	black
TG 51:2	0.079967	0.840683	black
TG 51:3	0.040864	0.924558	black
TG 52:1	0.178688	0.57749	black
TG 52:3	0.080253	0.720463	black
TG 53:2	0.074139	0.856672	black
TG 53:3	0.015531	0.955162	black
TG 54:3	0.240965	0.377308	black
TG 54:4	0.048911	0.880169	black
CAR 18:1	-0.07572	0.678849	black
CE 16:0	-0.00418	0.959245	black
CE 16:1	-0.31585	0.068471	black
CE 17:1	-0.09246	0.634887	black
CE 18:2	-0.05368	0.66671	black
CE 20:5	-0.30697	0.285732	black
CE 23:2	-0.01517	0.955162	black
Cer 40:1	-0.01021	0.955162	black
Cer 41:1	-0.09306	0.491478	black
Cer 42:1	-0.06759	0.581308	black
Cer 42:2	-0.15303	0.17026	black
Cer 42:3	-0.15604	0.277577	black
Chol 0:1	-0.07136	0.458379	black
DG 36:3	-0.10381	0.647101	black
DG 36:4	-0.2966	0.141295	black
LPC 14:0	-0.29558	0.056204	black
LPC 16:1	-0.13999	0.37949	black
LPC 20:3	-0.11337	0.428399	black
LPC 20:4	-0.07387	0.607786	black
LPC 22:6	-0.05484	0.730656	black
LPE 18:2	-0.29662	0.073586	black
PC-O 36:4	-0.07765	0.488867	black
PC-O 36:5	-0.11912	0.377308	black
PC-O 38:4	-0.02619	0.905189	black
PC-O 38:5	-0.14392	0.223072	black
PC-O 38:6	-0.10916	0.486672	black
PC-O 40:5	-0.09725	0.491478	black
PC-O 40:6	-0.01817	0.925011	black
PC 32:1	-0.26713	0.27552	black
PC 32:2	-0.11023	0.327292	black
PC 33:3	-0.12624	0.57749	black

PC 33:4	-0.06559	0.66671	black
PC 35:3	-0.32043	0.07491	black
PC 36:3	-0.10797	0.406123	black
PC 36:4	-0.14183	0.126935	black
PC 37:3	-0.3005	0.320579	black
PC 37:4	-0.10307	0.497729	black
PC 37:7	-0.15926	0.39385	black
PC 38:6	-0.16011	0.207491	black
PC 39:4	-0.1432	0.406123	black
PC 39:5	-0.06868	0.721141	black
PC 39:6	-0.33834	0.063198	black
PC 40:4	-0.10774	0.51652	black
PC 40:6	-0.29618	0.066246	black
PC 42:7	-0.17418	0.277577	black
PE-P 38:6	-0.19375	0.137202	black
PE 36:4	-0.06559	0.66671	black
PE 40:7	-0.15926	0.39385	black
SM 32:1	-0.08622	0.589642	black
SM 34:2	-0.09426	0.476349	black
SM 41:2	-0.0085	0.955162	black
TG 48:1	-0.06607	0.924158	black
TG 48:2	-0.15135	0.683383	black
TG 48:3	-0.23459	0.553665	black
TG 50:2	-0.10788	0.69153	black
TG 50:3	-0.21518	0.428399	black
TG 50:4	-0.47089	0.094163	black
TG 51:4	-0.09246	0.724766	black
TG 52:4	-0.0599	0.840683	black
TG 53:4	-0.16681	0.598609	black
TG 53:5	-0.2331	0.407893	black
TG 54:5	-0.28478	0.259051	black

Table S15: Log2-transformed FC in lipid abundances for MDD against SCZ for Cohort2. "color_condition" column depicts the significance of the lipid difference (two-sample two-sided t-test): "red" for BH-corrected $p < 0.05$, "black" for BH-corrected $p > 0.05$.

Lipid	logFC	BH-corrected P.value	color_condition
Cer 34:1	0.244492	5.22E-05	red
Cer 40:1	0.234156	0.000953	red
Cer 41:1	0.155995	0.028773	red
Cer 42:1	0.242972	0.000953	red
PC-O 34:3	0.178215	0.032401	red

PC-O 36:3	0.399832	0.005681	red
PC-O 36:6	0.322228	0.000566	red
PE-P 36:2	0.368354	0.003581	red
PE-P 36:4	0.193906	0.032401	red
SM 40:1	0.137168	0.046931	red
SM 42:1	0.175265	0.044887	red
CE 16:1	-0.34495	0.019523	red
CE 17:1	-0.35796	0.001833	red
Cer 42:3	-0.17794	0.024461	red
LPC 15:0	-0.25473	0.002268	red
LPC 16:0	-0.14459	0.035075	red
LPC 16:1	-0.32177	0.004184	red
LPC 17:0	-0.23164	0.010978	red
LPE 18:0	-0.25473	0.002268	red
PC-O 34:1	-0.17622	0.024818	red
PC-O 40:5	-0.26428	0.008207	red
PC 33:1	-0.47771	0.002785	red
PC 34:4	-0.26044	0.049955	red
PC 35:1	-0.63387	0.000566	red
PC 35:3	-0.26589	0.026672	red
PC 36:3	-0.18181	0.02335	red
PC 37:3	-0.58034	0.00177	red
PC 37:4	-0.35843	0.000307	red
PC 39:4	-0.40666	2.51E-05	red
PC 39:5	-0.27543	0.004184	red
PC 39:6	-0.2499	0.010978	red
PC 39:7	-0.20803	0.014107	red
PC 40:4	-0.36163	0.000953	red
PC 40:5	-0.41194	0.023264	red
PC 40:7	-0.16951	0.023264	red
PC 42:7	-0.36695	0.000386	red
PC 42:8	-0.22545	0.004184	red
SM 37:1	-0.31519	0.001833	red
SM 38:2	-0.28029	0.000386	red
SM 41:2	-0.17637	0.023264	red
SM 42:2	-0.15864	0.01356	red
TG 56:5	-0.32306	0.023264	red
TG 56:6	-0.2818	0.038805	red
TG 58:6	-0.42696	0.008066	red
TG 58:7	-0.41535	0.01356	red
CE 18:2	0.082761	0.162266	black
CE 20:5	0.16657	0.471357	black
Cer 42:2	0.009647	0.922948	black

DG 34:1	0.00781	0.96496	black
LPC 18:2	0.077848	0.610678	black
LPC 22:6	0.006525	0.963809	black
LPE 18:2	0.140297	0.348102	black
PC-O 34:2	0.120783	0.221097	black
PC-O 36:2	0.127146	0.159788	black
PC-O 36:5	0.062873	0.47093	black
PC-O 38:6	0.107463	0.191676	black
PC-O 38:7	0.070731	0.37951	black
PC-O 40:7	0.026015	0.818416	black
PC 33:3	0.026954	0.868166	black
PC 33:5	0.028161	0.868166	black
PC 34:2	0.014793	0.868166	black
PC 36:2	0.058629	0.471357	black
PC 36:5	0.090465	0.085025	black
PC 38:8	0.048181	0.836811	black
PE-P 38:6	0.140025	0.082554	black
PE 36:5	0.028161	0.868166	black
SM 41:1	0.044251	0.648606	black
TG 46:1	0.131074	0.799602	black
TG 48:1	0.025171	0.949646	black
TG 50:1	0.066009	0.836811	black
TG 51:1	0.081932	0.837941	black
TG 52:1	0.10733	0.648606	black
CAR 18:1	-0.09877	0.487601	black
CE 16:0	-0.05178	0.433588	black
CE 18:3	-0.12706	0.487601	black
CE 20:4	-0.15363	0.117698	black
CE 22:6	-0.02949	0.869014	black
CE 23:2	-0.01303	0.908459	black
Chol 0:1	-0.01019	0.892863	black
DG 34:2	-0.06468	0.729929	black
DG 36:2	-0.05268	0.799602	black
DG 36:3	-0.02336	0.90291	black
DG 36:4	-0.0165	0.924406	black
LPC 14:0	-0.24507	0.05517	black
LPC 18:0	-0.03113	0.818416	black
LPC 18:1	-0.11388	0.333713	black
LPC 20:3	-0.07061	0.565761	black
LPC 20:4	-0.1352	0.175762	black
LPC O-16:1	-0.10059	0.279454	black
PC-O 32:1	-0.09195	0.189347	black
PC-O 34:4	-0.00127	0.982413	black

PC-O 36:4	-0.01007	0.922948	black
PC-O 38:4	-0.0278	0.842065	black
PC-O 38:5	-0.04605	0.652928	black
PC-O 40:4	-0.19491	0.124337	black
PC-O 40:6	-0.05548	0.499135	black
PC-O 40:8	-0.02812	0.799602	black
PC 30:0	-0.15913	0.305147	black
PC 32:0	-0.11872	0.064993	black
PC 32:1	-0.28164	0.11553	black
PC 32:2	-0.01124	0.924406	black
PC 32:3	-0.11614	0.443364	black
PC 33:2	-0.11612	0.221097	black
PC 33:4	-0.13355	0.175762	black
PC 34:3	-0.12831	0.175762	black
PC 34:5	-0.01936	0.916785	black
PC 35:5	-0.00406	0.96496	black
PC 36:4	-0.11226	0.100468	black
PC 36:6	-0.0377	0.793444	black
PC 37:2	-0.16799	0.085034	black
PC 37:5	-0.00257	0.972054	black
PC 37:6	-0.10747	0.175762	black
PC 37:7	-0.04067	0.726043	black
PC 38:6	-0.05893	0.487601	black
PC 38:7	-0.08053	0.28611	black
PC 40:6	-0.17112	0.110146	black
PC 40:8	-0.11631	0.123104	black
PE 36:2	-0.11612	0.221097	black
PE 36:4	-0.13355	0.175762	black
PE 40:6	-0.10747	0.175762	black
PE 40:7	-0.04067	0.726043	black
SM 32:1	-0.03652	0.754875	black
SM 34:1	-0.0307	0.657476	black
SM 34:2	-0.0455	0.565761	black
SM 35:1	-0.13312	0.111531	black
SM 36:1	-0.12688	0.143214	black
SM 36:2	-0.16325	0.059718	black
SM 39:1	-0.01942	0.869014	black
SM 40:2	-0.05279	0.472378	black
TG 46:2	-0.03611	0.924406	black
TG 48:2	-0.13378	0.702896	black
TG 48:3	-0.19978	0.485432	black
TG 49:1	-0.09401	0.809866	black
TG 50:2	-0.11539	0.648606	black

TG 50:3	-0.18986	0.346087	black
TG 50:4	-0.24249	0.234309	black
TG 50:5	-0.28535	0.162266	black
TG 51:2	-0.3066	0.175762	black
TG 51:3	-0.32552	0.085264	black
TG 51:4	-0.22222	0.191676	black
TG 52:3	-0.0629	0.772119	black
TG 52:4	-0.05264	0.820003	black
TG 52:5	-0.18034	0.333713	black
TG 52:6	-0.2339	0.235284	black
TG 53:2	-0.2348	0.304473	black
TG 53:3	-0.2748	0.123104	black
TG 53:4	-0.28166	0.124337	black
TG 53:5	-0.06118	0.799602	black
TG 54:3	-0.00256	0.982413	black
TG 54:4	-0.02706	0.898529	black
TG 54:5	-0.05697	0.799602	black
TG 54:6	-0.05089	0.836811	black
TG 54:7	-0.14882	0.490555	black
TG 56:7	-0.2129	0.175762	black
TG 58:8	-0.35632	0.053131	black

Table S16. Number of males and females having SCZ or MDD in Cohort 1 (C1) or Cohort 2 (C2).

	**SCZ_C1**	**SCZ_C2**	**MDD_C1**	**MDD_C2**
**Males**	44	43	16	30
**Females**	41	57	19	55

Table S17: Log2-transformed FC in lipid abundances for MDD against SCZ separately for females and males of 71 significant lipid common for both cohorts in sex-mixed analysis.

	**Females**			**Males**		
**Lipid**	**LogFC1**	**LogFC2**	**meanFC**	**LogFC1**	**LogFC2**	**meanFC**
CE 18:3	-0.49412	-0.30343	-0.39877	-0.48557	0.158813	-0.16338
CE 20:4	-0.50862	-0.16166	-0.33514	-0.6191	-0.07417	-0.34663
CE 22:6	-0.71073	-0.12506	-0.4179	-1.00458	0.111536	-0.44652
CE 16:1	-0.35757	-0.61873	-0.48815	-0.29409	0.026477	-0.1338
CE 17:1	-0.16252	-0.5534	-0.35796	-0.01217	-0.10835	-0.06026
Cer 34:1	0.15789	0.311442	0.234666	-0.03652	0.149503	0.056492
Cer 40:1	-0.03838	0.11662	0.039121	0.019437	0.337157	0.178297
Cer 41:1	-0.17991	0.089033	-0.04544	-0.01885	0.164803	0.072976
Cer 42:1	-0.15059	0.138044	-0.00627	0.01488	0.398611	0.206746
Cer 42:3	-0.16364	-0.33806	-0.25085	-0.16757	0.026862	-0.07035

LPC 16:0	0.08299	-0.13025	-0.02363	0.100708	-0.13718	-0.01824
LPC 16:1	-0.14119	-0.39465	-0.26792	-0.13414	-0.1759	-0.15502
LPC 17:0	0.063496	-0.21558	-0.07604	0.209643	-0.31032	-0.05034
LPC 15:0	0.242017	-0.2763	-0.01714	0.357131	-0.22999	0.063569
LPE 18:0	0.242017	-0.2763	-0.01714	0.357131	-0.22999	0.063569
PC-O 36:3	0.055959	0.4702	0.26308	-0.02248	0.396934	0.187229
PC-O 40:5	-0.11113	-0.2864	-0.19876	-0.08521	-0.16568	-0.12545
PC-O 36:6	-0.37461	0.290081	-0.04226	-0.46025	0.483765	0.011758
PC-O 38:7	-1.07261	0.026611	-0.523	-1.23252	0.195911	-0.51831
PC-O 34:1	0.705621	-0.17303	0.266293	0.568224	-0.15762	0.205304
PC-O 40:8	-0.75664	-0.01374	-0.38519	-0.893	0.003501	-0.44475
PC-O 34:3	0.14759	0.165007	0.156298	0.120165	0.285417	0.202791
PC-O 40:7	-0.50088	-0.06784	-0.28436	-0.62519	0.19787	-0.21366
PC 35:1	-0.19375	-0.93787	-0.56581	0.253912	-0.16872	0.042598
PC 33:1	-0.09868	-0.73719	-0.41793	0.156291	-0.15397	0.001161
PC 40:4	-0.03169	-0.48475	-0.25822	-0.1632	-0.14989	-0.15655
PC 37:3	-0.19027	-0.70008	-0.44517	-0.37504	-0.53881	-0.45692
PC 37:4	-0.21892	-0.46527	-0.34209	0.012821	-0.21744	-0.10231
PC 39:4	-0.12561	-0.51639	-0.321	-0.16034	-0.26807	-0.21421
PC 39:5	-0.20854	-0.41411	-0.31133	0.106179	-0.05711	0.024533
PC 39:6	-0.42027	-0.43347	-0.42687	-0.26511	-0.05425	-0.15968
PC 40:5	0.152913	-0.35175	-0.09942	0.145228	-0.42837	-0.14157
PC 36:3	-0.0651	-0.2748	-0.16995	-0.14876	-0.07343	-0.1111
PC 38:7	-0.47169	-0.12627	-0.29898	-0.43826	0.019081	-0.20959
PC 36:5	-0.13948	0.01068	-0.0644	-0.17718	0.214505	0.018664
PC 36:6	-0.52718	-0.23158	-0.37938	-0.43869	0.260705	-0.08899
PC 37:5	-0.23155	-0.03873	-0.13514	-0.34649	0.005416	-0.17054
PC 37:6	-0.50436	-0.251	-0.37768	-0.53332	0.111847	-0.21074
PC 42:7	-0.02015	-0.41849	-0.21932	-0.33641	-0.26223	-0.29932
PC 38:8	-0.42404	-0.32294	-0.37349	-0.32464	0.709408	0.192386
PC 39:7	-0.62293	-0.30263	-0.46278	-0.45517	-0.11316	-0.28417
PC 40:7	-0.35396	-0.21344	-0.2837	-0.33365	-0.09208	-0.21286
PC 40:8	-0.51843	-0.20501	-0.36172	-0.4189	0.057838	-0.18053
PC 42:8	-0.91907	-0.27239	-0.59573	-0.86917	-0.16619	-0.51768
PC 34:2	0.187841	-0.05184	0.067999	0.155406	0.10807	0.131738
PC 32:3	-0.50205	-0.28144	-0.39175	-0.64143	0.225312	-0.20806
PC 35:3	-0.26656	-0.45738	-0.36197	-0.38843	-0.04391	-0.21617
PC 32:0	0.302311	-0.21074	0.045784	0.22151	0.067702	0.144606
PC 30:0	-0.55743	-0.38609	-0.47176	-0.65017	0.300453	-0.17486
PC 34:5	-0.54138	-0.14116	-0.34127	-0.32955	0.256862	-0.03635
PC 34:4	-0.4688	-0.47955	-0.47417	-0.43603	0.134165	-0.15093
PE-P 36:4	0.044718	0.054895	0.049806	0.214961	0.486961	0.350961
PE-P 36:2	1.103675	0.429697	0.766686	1.437264	0.338111	0.887687

PE 40:6	-0.50436	-0.251	-0.37768	-0.53332	0.111847	-0.21074
SM 38:2	0.179683	-0.30802	-0.06417	0.047009	-0.2558	-0.10439
SM 37:1	0.021662	-0.44636	-0.21235	0.290711	-0.31114	-0.01021
SM 41:2	-0.00143	-0.26463	-0.13303	-0.05573	-0.11838	-0.08706
SM 42:2	-0.00929	-0.31325	-0.16127	0.128285	0.127806	0.128045
SM 42:1	0.053679	0.090357	0.072018	0.232425	0.416446	0.324436
SM 40:1	0.123229	0.009914	0.066571	0.307294	0.370415	0.338854
TG 58:7	-1.50519	-0.79081	-1.148	-1.47085	0.26897	-0.60094
TG 58:6	-1.76016	-0.73092	-1.24554	-1.82028	0.121226	-0.84953
TG 56:7	-1.44272	-0.5883	-1.01551	-1.07444	0.419565	-0.32744
TG 56:5	-1.11946	-0.59426	-0.85686	-0.88095	0.13501	-0.37297
TG 54:7	-1.33879	-0.68742	-1.01311	-0.63856	0.767863	0.06465
TG 54:6	-0.93417	-0.39122	-0.6627	-0.21354	0.464984	0.12572
TG 52:6	-1.14743	-0.68269	-0.91506	-0.40973	0.531719	0.060994
TG 52:5	-0.91918	-0.52639	-0.72279	-0.12244	0.35566	0.11661
TG 50:5	-1.05128	-0.66574	-0.85851	-0.13771	0.365442	0.113867
TG 58:8	-2.01322	-0.78672	-1.39997	-2.19019	0.392047	-0.89907
TG 56:6	-1.267	-0.56057	-0.91378	-0.99239	0.211206	-0.39059

Table S18: Log2-transformed FC in lipid abundances for MDD against SCZ for separate statistical analysis of females and males. Non-sign means that this lipid is not statistically significant for this group.

	**Males**		**Females**		
Lipid	**LogFC_1**	**LogFC_2**	**LogFC_1**	**LogFC_2**	**Class**
CE 20:4	-0.6191	-0.07417	-0.50862	-0.16166	CE
CE 22:6	-1.00458	0.111536	**Non-sign**	**Non-sign**	CE
LPC 15:0	0.357131	-0.22999	0.242017	-0.2763	LPC
LPE 18:0	0.357131	-0.22999	0.242017	-0.2763	LPE
PC-O 34:1	0.568224	-0.15762	0.705621	-0.17303	PC-O
PC-O 38:7	-1.23252	0.195911	-1.07261	0.026611	PC-O
PC-O 40:7	-0.62519	0.19787	**Non-sign**	**Non-sign**	PC-O
PC-O 40:8	-0.893	0.003501	-0.75664	-0.01374	PC-O
PC 32:3	-0.64143	0.225312	-0.50205	-0.28144	PC
PC 37:6	-0.53332	0.111847	-0.50436	-0.251	PC
PC 38:7	-0.43826	0.019081	-0.47169	-0.12627	PC
PC 39:7	-0.45517	-0.11316	-0.62293	-0.30263	PC
PC 42:8	-0.86917	-0.16619	-0.91907	-0.27239	PC
PE-P 36:2	1.437264	0.338111	1.103675	0.429697	PE-P
PE 40:6	-0.53332	0.111847	-0.50436	-0.251	PE
SM 40:1	0.307294	0.370415	**Non-sign**	**Non-sign**	SM
TG 56:5	-0.88095	0.13501	-1.11946	-0.59426	TG
TG 56:6	-0.99239	0.211206	-1.267	-0.56057	TG
TG 56:7	-1.07444	0.419565	-1.44272	-0.5883	TG

TG 58:6	-1.82028	0.121226	-1.76016	-0.73092	TG
TG 58:7	-1.47085	0.26897	-1.50519	-0.79081	TG
TG 58:8	-2.19019	0.392047	-2.01322	-0.78672	TG
CE 18:3	**Non-sign**	**Non-sign**	-0.49412	-0.30343	CE
PC 30:0	**Non-sign**	**Non-sign**	-0.55743	-0.38609	PC
PC 34:2	**Non-sign**	**Non-sign**	0.187841	-0.05184	PC
PC 34:4	**Non-sign**	**Non-sign**	-0.4688	-0.47955	PC
PC 34:5	**Non-sign**	**Non-sign**	-0.54138	-0.14116	PC
PC 36:6	**Non-sign**	**Non-sign**	-0.52718	-0.23158	PC
PC 38:8	**Non-sign**	**Non-sign**	-0.42404	-0.32294	PC
PC 40:7	**Non-sign**	**Non-sign**	-0.35396	-0.21344	PC
PC 40:8	**Non-sign**	**Non-sign**	-0.51843	-0.20501	PC
TG 50:4	**Non-sign**	**Non-sign**	-0.88701	-0.63602	TG
TG 50:5	**Non-sign**	**Non-sign**	-1.05128	-0.66574	TG
TG 52:5	**Non-sign**	**Non-sign**	-0.91918	-0.52639	TG
TG 52:6	**Non-sign**	**Non-sign**	-1.14743	-0.68269	TG
TG 54:5	**Non-sign**	**Non-sign**	-0.57854	-0.30387	TG
TG 54:6	**Non-sign**	**Non-sign**	-0.93417	-0.39122	TG
TG 54:7	**Non-sign**	**Non-sign**	-1.33879	-0.68742	TG
CE 16:1	**Non-sign**	**Non-sign**	-0.35757	-0.61873	CE
CE 17:1	**Non-sign**	**Non-sign**	-0.16252	-0.5534	CE
Cer 34:1	**Non-sign**	**Non-sign**	0.15789	0.311442	Cer
Cer 42:3	**Non-sign**	**Non-sign**	-0.16364	-0.33806	Cer
LPC 14:0	**Non-sign**	**Non-sign**	-0.3648	-0.32449	LPC
LPC 16:1	**Non-sign**	**Non-sign**	-0.14119	-0.39465	LPC
PC-O 32:1	**Non-sign**	**Non-sign**	0.223582	-0.14697	PC-O
PC-O 36:3	**Non-sign**	**Non-sign**	0.055959	0.4702	PC-O
PC-O 36:6	**Non-sign**	**Non-sign**	-0.37461	0.290081	PC-O
PC-O 40:5	**Non-sign**	**Non-sign**	-0.11113	-0.2864	PC-O
PC 32:0	**Non-sign**	**Non-sign**	0.302311	-0.21074	PC
PC 32:1	**Non-sign**	**Non-sign**	-0.33456	-0.60909	PC
PC 33:1	**Non-sign**	**Non-sign**	-0.09868	-0.73719	PC
PC 33:2	**Non-sign**	**Non-sign**	0.041931	-0.25773	PC
PC 33:4	**Non-sign**	**Non-sign**	-0.11202	-0.34546	PC
PC 34:3	**Non-sign**	**Non-sign**	-0.05021	-0.30415	PC
PC 35:1	**Non-sign**	**Non-sign**	-0.19375	-0.93787	PC
PC 35:3	**Non-sign**	**Non-sign**	-0.26656	-0.45738	PC
PC 36:3	**Non-sign**	**Non-sign**	-0.0651	-0.2748	PC
PC 36:4	**Non-sign**	**Non-sign**	-0.18946	-0.21647	PC
PC 37:2	**Non-sign**	**Non-sign**	0.114309	-0.24309	PC
PC 37:3	**Non-sign**	**Non-sign**	-0.19027	-0.70008	PC
PC 37:4	**Non-sign**	**Non-sign**	-0.21892	-0.46527	PC
PC 37:7	**Non-sign**	**Non-sign**	-0.18545	-0.21748	PC

PC 38:6	**Non-sign**	**Non-sign**	-0.17516	-0.20617	PC
PC 39:4	**Non-sign**	**Non-sign**	-0.12561	-0.51639	PC
PC 39:5	**Non-sign**	**Non-sign**	-0.20854	-0.41411	PC
PC 39:6	**Non-sign**	**Non-sign**	-0.42027	-0.43347	PC
PC 40:4	**Non-sign**	**Non-sign**	-0.03169	-0.48475	PC
PC 40:6	**Non-sign**	**Non-sign**	-0.28689	-0.38458	PC
PC 42:7	**Non-sign**	**Non-sign**	-0.02015	-0.41849	PC
PE 36:2	**Non-sign**	**Non-sign**	0.041931	-0.25773	PE
PE 36:4	**Non-sign**	**Non-sign**	-0.11202	-0.34546	PE
PE 40:7	**Non-sign**	**Non-sign**	-0.18545	-0.21748	PE
SM 35:1	**Non-sign**	**Non-sign**	0.043823	-0.26277	SM
SM 36:1	**Non-sign**	**Non-sign**	0.016296	-0.27736	SM
SM 36:2	**Non-sign**	**Non-sign**	0.04277	-0.28552	SM
SM 37:1	**Non-sign**	**Non-sign**	0.021662	-0.44636	SM
SM 38:2	**Non-sign**	**Non-sign**	0.179683	-0.30802	SM
SM 41:2	**Non-sign**	**Non-sign**	-0.00143	-0.26463	SM
SM 42:2	**Non-sign**	**Non-sign**	-0.00929	-0.31325	SM
TG 48:2	**Non-sign**	**Non-sign**	-0.46542	-0.58828	TG
TG 48:3	**Non-sign**	**Non-sign**	-0.64152	-0.60272	TG
TG 50:2	**Non-sign**	**Non-sign**	-0.38492	-0.51727	TG
TG 50:3	**Non-sign**	**Non-sign**	-0.54257	-0.5909	TG
TG 51:2	**Non-sign**	**Non-sign**	-0.22678	-0.71892	TG
TG 51:3	**Non-sign**	**Non-sign**	-0.30814	-0.69881	TG
TG 51:4	**Non-sign**	**Non-sign**	-0.4098	-0.55758	TG
TG 52:3	**Non-sign**	**Non-sign**	-0.18973	-0.35216	TG
TG 53:2	**Non-sign**	**Non-sign**	-0.19828	-0.60912	TG
TG 53:3	**Non-sign**	**Non-sign**	-0.26601	-0.59778	TG
TG 53:4	**Non-sign**	**Non-sign**	-0.52394	-0.63656	TG

Table S19. 20 lipids from the discordant clusters intersected with statistical analysis MDD-SCZ (MDD-SCZ lipid panel).

**Cluster 1**	**Cluster 2**
CE 17:1	PC 42:8
CE 18:3	TG 52:6
Cer 42:3	TG 54:7
LPC 16:1	TG 56:5
PC 30:0	TG 56:6
PC 32:3	TG 56:7
PC 34:4	TG 58:6
PC 37:3	TG 58:7
PC 40:4	TG 58:8
PC 42:7	

TG 50:5	

Table S20. Results of Kolmogorov-Smirnov (KS) test for PC1 density distribution in both cohorts (PCA analysis using 20 species from MDD-SCZ lipid panel). Compared groups represent the distribution of groups for which the particular test was performed. C1 stands for Cohort 1 and C2 stands for Cohort 2.

**Compared groups**		**KS Statistic**	**p-value**	**BH-p_adjusted**
SCZ_C1	MDD_C1	0.6471	2.14E-10	4.49E-09
SCZ_C1	TYP_C1	0.1072	0.8024	0.8869
SCZ_C1	AFF_C1	0.1627	0.540774	0.7571
MDD_C1	TYP_C1	0.6659	1.77E-09	1.24E-08
MDD_C1	AFF_C1	0.5667	2.52E-05	8.81E-05
TYP_C1	AFF_C1	0.1756	0.5302	0.7571
SCZ_C2	MDD_C2	0.2900	0.0006	0.0019
SCZ_C2	BPD_C2	0.3331	0.0099	0.0190
MDD_C2	BPD_C2	0.1753	0.4604	0.7438

Table S21. Metrics of SCZ-CTR logistic regression classification model for training and test datasets.

**Training set**				
	**Precision**	**Recall**	**F1-score**	**Sample number**
0 (CTR)	0.95	0.98	0.97	60
1 (SCZ)	0.99	0.97	0.98	102
Accuracy			0.98	
Macro average	0.97	0.98	0.97	
Weighted average	0.98	0.98	0.98	
**Test set**				
	**Precision**	**Recall**	**F1-score**	**Sample number**
0 (CTR)	0.89	0.8	0.84	20
1 (SCZ)	0.85	0.92	0.88	24
Accuracy			0.86	
Macro average	0.87	0.86	0.86	
Weighted average	0.87	0.86	0.86	

Table S22. Metrics of MDD-CTR logistic regression classification model for training and test datasets.

**Training set**				
	**Precision**	**Recall**	**F1-score**	**Sample number**

0 (CTR)	1	1	1	60
1 (MDD)	1	1	1	63
Accuracy			1	
Macro average	1	1	1	
Weighted average	1	1	1	
**Test set**				
	**Precision**	**Recall**	**F1-score**	**Sample number**
0 (CTR)	0.95	0.9	0.92	20
1 (MDD)	0.89	0.94	0.91	17
Accuracy			0.92	
Macro average	0.92	0.92	0.92	
Weighted average	0.92	0.92	0.92	

Table S23. Metrics of MDD-SCZ logistic regression classification model for training and test datasets.

**Training set**				
	**Precision**	**Recall**	**F1-score**	**Sample number**
0 (SCZ)	0.95	1	0.98	102
1 (MDD)	1	0.92	0.96	63
Accuracy			0.97	
Macro average	0.98	0.96	0.97	
Weighted average	0.97	0.97	0.97	
**Test set**				
	**Precision**	**Recall**	**F1-score**	**Sample number**
0 (SCZ)	0.75	0.88	0.81	24
1 (MDD)	0.77	0.59	0.67	17
Accuracy			0.76	
Macro average	0.76	0.73	0.74	
Weighted average	0.76	0.76	0.75	

Table S24. Metrics of MDD-SCZ logistic regression classification model for cohort 1 or cohort 2 only used as test set.

**Test: Cohort 1**				
	**Precision**	**Recall**	**F1-score**	**Sample number**
0 (SCZ)	1	0.89	0.94	45
1 (MDD)	0.67	1	0.8	10
Accuracy			0.91	
Macro average	0.83	0.94	0.87	
Weighted average	0.94	0.91	0.92	
**Test: Cohort 2**				
	**Precision**	**Recall**	**F1-score**	**Sample number**
0 (SCZ)	0.54	0.71	0.61	38
1 (MDD)	0.69	0.51	0.59	47
Accuracy			0.6	
Macro average	0.61	0.61	0.6	
Weighted average	0.62	0.6	0.6	

Table S25. Intersection of lipids with non-zero coefficient in the constructed logistic regression and 20 species from MDD-SCZ lipid panel (Table S19).

Cer 42:3	LPC 16:1	PC 30:0	PC 37:3	PC 40:4	PC 42:7	PC 42:8	TG 58:6
